# Deletion of IRE1α in podocytes exacerbates diabetic nephropathy in mice

**DOI:** 10.1038/s41598-024-62599-7

**Published:** 2024-05-22

**Authors:** Andrey V. Cybulsky, Joan Papillon, Julie Guillemette, José R. Navarro-Betancourt, Chen-Fang Chung, Takao Iwawaki, I. George Fantus

**Affiliations:** 1 Department of Medicine, McGill University Health Centre Research Institute, McGill University, Montreal, QC Canada; 2 Department of Life Science, Kanazawa Medical University, Uchinada, Japan

**Keywords:** Albuminuria, Autophagy, Endoplasmic reticulum, Gene expression, Glomerulopathy, Unfolded protein response, Nephrology, Kidney, Kidney diseases

## Abstract

Protein misfolding in the endoplasmic reticulum (ER) of podocytes contributes to the pathogenesis of glomerular diseases. Protein misfolding activates the unfolded protein response (UPR), a compensatory signaling network. We address the role of the UPR and the UPR transducer, inositol-requiring enzyme 1α (IRE1α), in streptozotocin-induced diabetic nephropathy in mice. Diabetes caused progressive albuminuria in control mice that was exacerbated in podocyte-specific IRE1α knockout (KO) mice. Compared to diabetic controls, diabetic IRE1α KO mice showed reductions in podocyte number and synaptopodin. Glomerular ultrastructure was altered only in diabetic IRE1α KO mice; the major changes included widening of podocyte foot processes and glomerular basement membrane. Activation of the UPR and autophagy was evident in diabetic control, but not diabetic IRE1α KO mice. Analysis of human glomerular gene expression in the JuCKD-Glom database demonstrated induction of genes associated with the ER, UPR and autophagy in diabetic nephropathy. Thus, mice with podocyte-specific deletion of IRE1α demonstrate more severe diabetic nephropathy and attenuation of the glomerular UPR and autophagy, implying a protective effect of IRE1α. These results are consistent with data in human diabetic nephropathy and highlight the potential for therapeutically targeting these pathways.

## Introduction

Human glomerular diseases, including primary glomerulopathies and diabetic nephropathy (DN) are leading causes of chronic kidney disease, and have a major impact on health^1^. Current therapies of glomerulopathies and diabetic nephropathy are only partially effective and lack specificity; furthermore, certain treatments of glomerulonephritis induce side effects^1^. Thus, the goal is to develop mechanism-based therapies.

Podocytes or glomerular visceral epithelial cells (GECs), mesangial and endothelial cells may all be involved in the pathogenesis of glomerulonephritis and diabetic nephropathy. Among these cells, podocytes are highly-differentiated epithelial cells that are vital in maintaining glomerular capillary wall permselectivity^2,3^, and podocyte injury is believed to be key to the pathogenesis of many glomerular diseases. The pathogenic mechanisms that initiate the various glomerulopathies differ markedly from one another; however, podocyte injury in these diseases features impaired protein folding and protein homeostasis (“proteostasis”) within the endoplasmic reticulum (ER)^4^. This damages the glomerulus and drives proteinuria in glomerulopathies^4,5^. Thus, maintenance of proteostasis is critical to preserving integrity of podocytes—long-living cells with limited turnover capacity^2,3^.

Newly translated proteins destined for secretion or to the plasma membrane (the “secretory pathway”) attain their proper folding conformations with the help of chaperones in the ER. Folded proteins are trafficked from the ER to the Golgi to undergo further post-translational modifications^4,6,7^. Intact ER function is important for proteostasis in podocytes, including production of components of the slit diaphragm, focal adhesion complexes and glomerular basement membrane (GBM)^2,3^. Disruption of ER function causes accumulation of misfolded proteins, ER stress and activation of the unfolded protein response (UPR)^4,7–9^. The UPR is an *adaptive* mechanism that maintains ER function, enhances protein folding and alleviates stress caused by the misfolded proteins^7,8^. However, prolonged ER stress that causes chronic over-activation of the UPR can be cytotoxic and can induce apoptosis^7,8^. There are three UPR transducers; each one mediates one of three UPR signaling axes: activating transcription factor 6 (ATF6), PKR-like ER kinase (PERK) and inositol-requiring enzyme 1α (IRE1α). Our focus is on IRE1α, a protein kinase and RNase, and the most evolutionarily conserved UPR transducer. Classically, IRE1α RNase splices X-box binding protein 1 (XBP1) mRNA to generate a potent transcriptional activator of ER chaperone genes (sXBP1), which leads to enhanced ER protein folding capacity. Proteins that remain misfolded in the ER despite UPR activation can be removed via ER-associated degradation^6,7^. In addition, there is emerging evidence that macroautophagy (hereafter referred to as autophagy) is linked with IRE1α and the UPR, and autophagy is an important protective mechanism to clear misfolded proteins in podocytes^4,5,9–12^. Thus, the UPR with its linkage to autophagy are especially critical in maintaining proteostasis in podocytes.

There is evidence for ER stress in human glomerulopathies—ER stress markers are increased in the glomeruli of kidney biopsies from patients with glomerulonephritis and diabetic nephropathy^4,13,14^. Analysis of a human glomerular gene expression dataset (Nephroseq) supports activation of a UPR-IRE1α axis in patients with various glomerulopathies^15,16^. We demonstrated the functional importance of the IRE1α-UPR axis using mice with podocyte-specific deletion of IRE1α (IRE1α knockout; KO mice)^17^. Male IRE1α KO mice developed albuminuria (starting at 5 months of age and worsening until 13 months), podocyte depletion, and ultrastructural podocyte injury. Thus, IRE1α was protective under basal conditions, suggesting that activation of the UPR is adaptive in normal aging. In the classic adriamycin mouse model of human focal segmental glomerulosclerosis (FSGS), where adriamycin induces podocyte injury and nephrosis, there is protein misfolding in the ER, and activation of the glomerular UPR and autophagy. Podocyte deletion of IRE1α exacerbated podocyte injury in adriamycin nephrosis, as well as in anti-GBM nephritis^15,17^. Adriamycin-treated male mice with IRE1α KO show ~ 20-fold greater albuminuria, severe podocyte foot process effacement, markedly dilated ER and striking mitochondrial damage compared to adriamycin-treated control mice^15^. These studies indicate that in response to injury, activation of IRE1α preserves glomerular integrity in preclinical and most likely human glomerulonephritis.

The pathogenesis of diabetic nephropathy is complex and poorly understood^18–20^. Briefly, hyperglycemia and oxidative stress stimulate diacylglycerol, protein kinase C, the polyol pathway and advanced glycation end products (e.g. protein glycation), leading to stimulation of inflammatory mediators/cytokines and growth factors. This results in renal hemodynamic changes and structural damage, including podocyte injury. Indeed, podocyte injury is key in the pathogenesis of diabetic nephropathy^12,18,21,22^. A widely used experimental model of type 1 diabetes is induced by streptozotocin (STZ), a chemical toxin for pancreatic β-cells. Kidney injury resembles human diabetic nephropathy, and includes albuminuria, podocyte loss, mesangial and GBM expansion, decline in renal function and eventually glomerular and tubulointestitial sclerosis, although a limitation of the model is that injury is relatively mild^20,21,23^. There has been considerable interest in the role of ER stress, the UPR and autophagy in the pathogenesis of experimental diabetic nephropathy^10,12,22,24,25^, but conflicting results on their mechanistic roles have been reported, and these require more precise definition. In the present study, we demonstrate that diabetic nephropathy in mice is associated with albuminuria, podocyte injury and activation of the glomerular UPR and autophagy. Mice with podocyte-specific deletion of IRE1α demonstrate more severe diabetic nephropathy, and attenuation of the glomerular UPR and autophagy, indicating a protective mechanism mediated via IRE1α.

## Materials and methods

### Antibodies and chemicals

Rabbit antibodies to LC3B (2775) and SQSTM1/p62 (5114) were purchased from Cell Signaling Technology (Danvers, MA). Goat anti-synaptopodin (sc-21537) and rat anti-GRP94 (sc-32249) antibodies were from Santa Cruz Biotechnology (Santa Cruz, CA). Rabbit anti-Wilms tumor-1 (WT1; CAN-R9(IHC)-56-2; ab89901) was from Abcam Inc. (Toronto, ON). Rabbit anti-mesencephalic astrocyte-derived neurotrophic factor (MANF; PAB13301) was from Abnova (Walnut, CA). Rat anti-collagen α5 (IV) clone H53 (7078) was purchased from Chondrex Inc. (Woodinville, WA). Mouse anti-LC3B clone 5F10 (ALX-830-080-C100) was from Enzo Life Sciences (Ann Arbor, MI). Rabbit anti-peroxisome proliferator-activated receptor gamma coactivator 1-α (PGC1α; PA5-38021) was purchased from Thermo-Fisher Scientific (Burlington, ON). Rabbit anti-actin (A2066) was from MilliporeSigma (Mississauga, ON). Rabbit anti-nephrin antiserum was a gift from Dr. Tomoko Takano (McGill University)^15^. Non-immune IgG and secondary antibodies were from Jackson ImmunoResearch Laboratories (West Grove, PA) or Thermo-Fisher Scientific. Streptozotocin (STZ) and rhodamine-phalloidin were from MilliporeSigma.

### Studies in mice

Generation, genotyping and characterization of podocyte-specific IRE1α KO mice was described previously^15,17^. Briefly, mice with loxP sites surrounding exons 20–21 (RNAse domain) of IRE1α gene were bred with mice expressing a Cre recombinase under control of the podocyte-specific podocin promoter. This deletes exons 20–21 in podocytes. The mutant IRE1α protein is not detectable in vivo, indicating that it is most likely unstable and degraded. The expression of IRE1α protein is reduced in glomerular lysates of IRE1α knockout mice, compared with littermate controls^15,17^. The animals were housed in a pathogen free facility, under standard conditions including cages and bedding, with 12 h on–off light cycles, and were fed ad libitum. Adult (3–4 month old) male control and IRE1α KO littermates were untreated or received STZ 50 mg/kg (in 50 mM sodium citrate buffer, pH 4.5) intraperitoneally for 5 days^23^. Mice were given 10% sucrose water to drink for 6 days after STZ treatment. The animals were randomly allocated to experimental groups. Blood glucose was measured after 1 week using glucose test strips. If hyperglycemia did not develop, the protocol, was repeated. This low-dose STZ protocol avoids direct STZ renal toxicity. Mice were followed for 6 months and were euthanized (isofluorane followed by cervical dislocation) in the animal facility to collect the kidneys and isolate glomeruli by sequential sieving^17,26^. The animal protocol was approved by the McGill University Animal Care Committee, and our studies comply with the guidelines established by the Canadian Council on Animal Care. The study complied with ARRIVE guidelines.

Spot urine samples were collected in the morning in the animal facility at monthly intervals until the mice were euthanized. Urine albumin was quantified with an enzyme-linked immunosorbent assay (Mouse Albumin ELISA Quantification Kit, Bethyl Laboratories, Montgomery, TX). Albumin results were normalized to urine creatinine, which was measured using a picric acid-based reaction (Creatinine Colorimetric Assay Kit, Cayman Chemical Co; Ann Arbor, MI).

### Studies in cell culture

Primary-immortalized non-clonal control and IRE1α KO GECs were generated and characterized as described previously^15^. In KO GECs, there is an absence of wild type IRE1α mRNA and protein. GECs were cultured in K1 medium (Supplementary Table 1)^15^ in a 7.8 mM glucose concentration. For experiments, GECs were allowed to proliferate for one day at 33 °C, and were then switched to 37 °C to differentiate for 48 h.

### Microscopy

For light microscopy, portions of kidneys were fixed in 4% paraformaldehyde and stained with periodic acid-Schiff by conventional techniques at the McGill University Health Centre Histology Platform. Quantitative morphometry was used to characterize histological changes objectively (i.e. minimize observer bias). Slides were digitized at 40 × resolution in an Aperio AT Turbo scanner (Leica Biosystems, Buffalo Grove, IL). Images were processed using Aperio ImageScope 12.4 (Leica Biosystems). Glomeruli in experimental groups were selected randomly and analyzed with the Positive Pixel Count v9 algorithm, as reported previously^15,27^. Positive pixels were identified by a hue value of 0.854 (pink) and a hue width of 0.035. Glomerular matrix expansion was expressed as the ratio of positive over total pixels. For WT1 counts, kidney sections were deparaffinized and rehydrated. Antigen retrieval was done using citrate buffer, pH 6. Automated immunohistochemistry staining was performed by the McGill University Health Centre Histology Platform, using the Discovery Ultra Instrument (Roche Diagnostics). Cell nuclei were then quantified by visual counting.

For immunofluorescence microscopy, kidney poles were snap-frozen in isopentane (− 80 °C). Cryostat sections (4 μm thickness) were cut and then fixed in 4% paraformaldehyde (22 °C), ice-cold methanol or ice-cold acetone, and blocked with 5% normal rabbit or goat serum or 3–5% BSA. Incubations with primary antibodies were performed overnight at 4 °C, and incubations with secondary antibodies were 1 h at 22 °C. In control incubations (performed in parallel), primary antibody was replaced with nonimmune IgG. In some experiments, cell nuclei were stained with Hoechst H33342^15,28^. Glomeruli in experimental groups were selected randomly and images were acquired using a Zeiss Axio Observer Z1 LSM780 laser scanning confocal microscope with ZEN2010 software (McGill University Health Centre Research Institute Imaging Platform). To compare fluorescence intensities, all images were taken at the same exposure time. Fluorescence intensity was quantified using the histogram function of ImageJ software (National Institutes of Health, Bethesda, MD), and results are expressed in arbitrary units^15,28^. The glomerular fluorescence intensity was normalized to the total fluorescence in each image. The ImageJ protocol to quantify LC3 puncta was detailed previously^28,29^. To measure the colocalization of two proteins in mouse kidney sections, glomeruli were circled, and threshold intensity of each channel was measured, as previously. Colocalization of the thresholded images in kidney sections was determined using the JACoP plugin in ImageJ^28,30^.

For transmission electron microscopy, kidney sections were fixed with 2.5% glutaraldehyde in 0.1 M sodium cacodylate buffer and processed at the McGill University Facility for Electron Microscopy Research. Tissues were imaged with a Tecnai 12 electron microscope linked to an AMTV601 CCD camera (Field Electron and Ion Company, Hillsboro, OR). Podocyte foot process and GBM width were measured and calculated, as described previously^15,17^. The number of foot processes was counted along a given length of GBM in a capillary loop. Foot process width was calculated according to the formula (π × GBM length)/(4 × foot process number). GBM width was measured perpendicularly in 4 places at comparably-spaced intervals along each glomerular capillary loop.

### Immunoblotting

The protocol for immunoblotting was described earlier^17^. Chemiluminescence was detected in a ChemiDoc Touch Imaging System (Bio-Rad; Mississauga, ON); signal saturation was monitored with Image Lab (Bio-Rad) and only signal intensities within a linear range were analyzed. Band densitometries were quantified using ImageJ and values were normalized to the expression of β-actin.

### Nephroseq dataset analysis

The publicly accessible Nephroseq dataset “JuCKD-Glom” (GSE47183) was used for the expression analysis of glomerular genes, including genes associated with the ER/UPR and autophagy^16,31,32^. Nephroseq contains microarray gene expression data (mRNAs) of microdissected whole glomeruli from human kidney biopsies and the data are presented as the fold-increase of gene expression in disease above healthy control. The P-values and P-values adjusted for the false discovery rate (i.e. Q-values) for the full dataset, which we present in this manuscript, are those calculated and reported within the datasets by their original authors. P-values for ER/UPR and autophagy genes were corrected for false discovery according to the Benjamini–Hochberg method. Pathway overrepresentation and gene ontology (GO) enrichment analyses were performed using the ConsensusPathDB interaction database^16,33^. This database provides the adjusted P-values (hypergeometric test, corrected for multiple comparisons) that we present in this manuscript. Pathway overrepresentation and gene ontology (GO) enrichment analyses was also performed using a second separate dataset of glomerular gene expression in patients with diabetic nephropathy and normal controls (GSE 30122)^34^.

### Statistical analysis

Values are expressed as mean ± standard error. Data were processed in Prism (GraphPad Software, La Jolla, CA). In experiments with three or more groups, or groups and multiple time-points, one way or two-way analysis of variance (ANOVA) was used to determine significant differences among groups; where relevant, additional comparisons were calculated and values were adjusted according to the Holm-Sidak method. Significant differences between two groups are displayed with lines between columns, with asterisks denoting P values. In the absence of such lines, differences were not statistically significant.

## Results

### Deletion of IRE1α in podocytes exacerbates albuminuria and podocyte loss in diabetic nephropathy

Mice with podocyte-specific deletion of IRE1α (age 3–4 months) were used to address the functional role of IRE1α in STZ-induced diabetic nephropathy. Within one month after treatment of mice with STZ, a major increase in blood glucose was evident in both control and podocyte-specific IRE1α KO mice, indicating development of diabetes (Supplementary Fig. 1). Diabetic IRE1α KO mice demonstrated 7–17% weight loss compared with untreated KO mice over 6 months (Supplementary Fig. 1).

**Figure 1:**
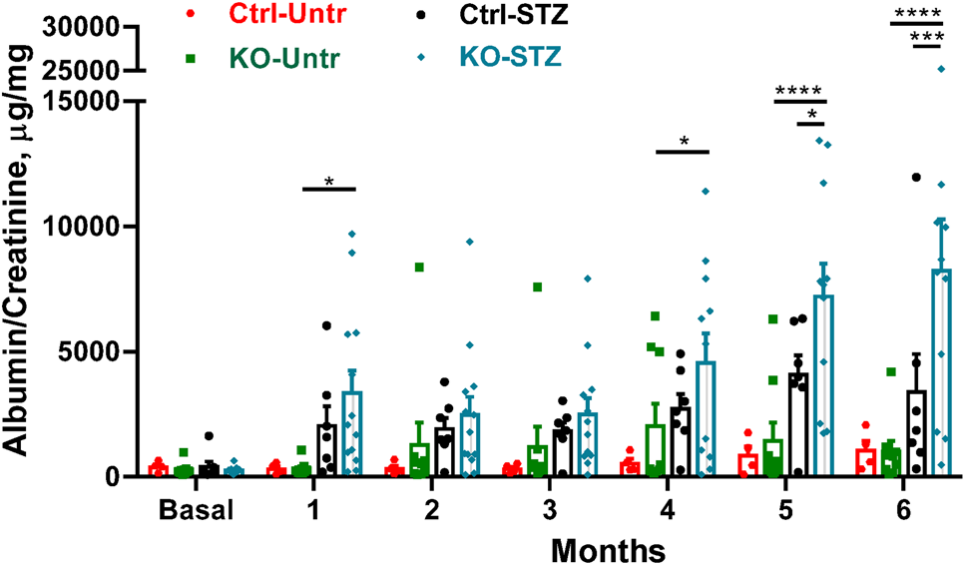
Podocyte-specific deletion of IRE1α exacerbates albuminuria in diabetic nephropathy. Induction of diabetes with STZ resulted in progressive albuminuria in IRE1α KO mice. N = 4 mice in control (Ctrl) untreated (Untr), 9 in KO Untr, 7 in Ctrl STZ and 13 in KO STZ groups. In the KO STZ group, 2 animals died prior to 6 months and are included until the time-points of death. In the other 3 groups, data on all animals are up to 6 months. Data were analyzed with a 2-way ANOVA. When the 1–6 month time points are considered together, significant changes are: P < 0.01 Ctrl STZ vs KO STZ and P < 0.0001 KO Untr vs KO STZ. Ctrl Untr vs Ctrl STZ did not reach statistical significance. *P < 0.05, ***P < 0.001, ****P < 0.0001.

**Figure 2:**
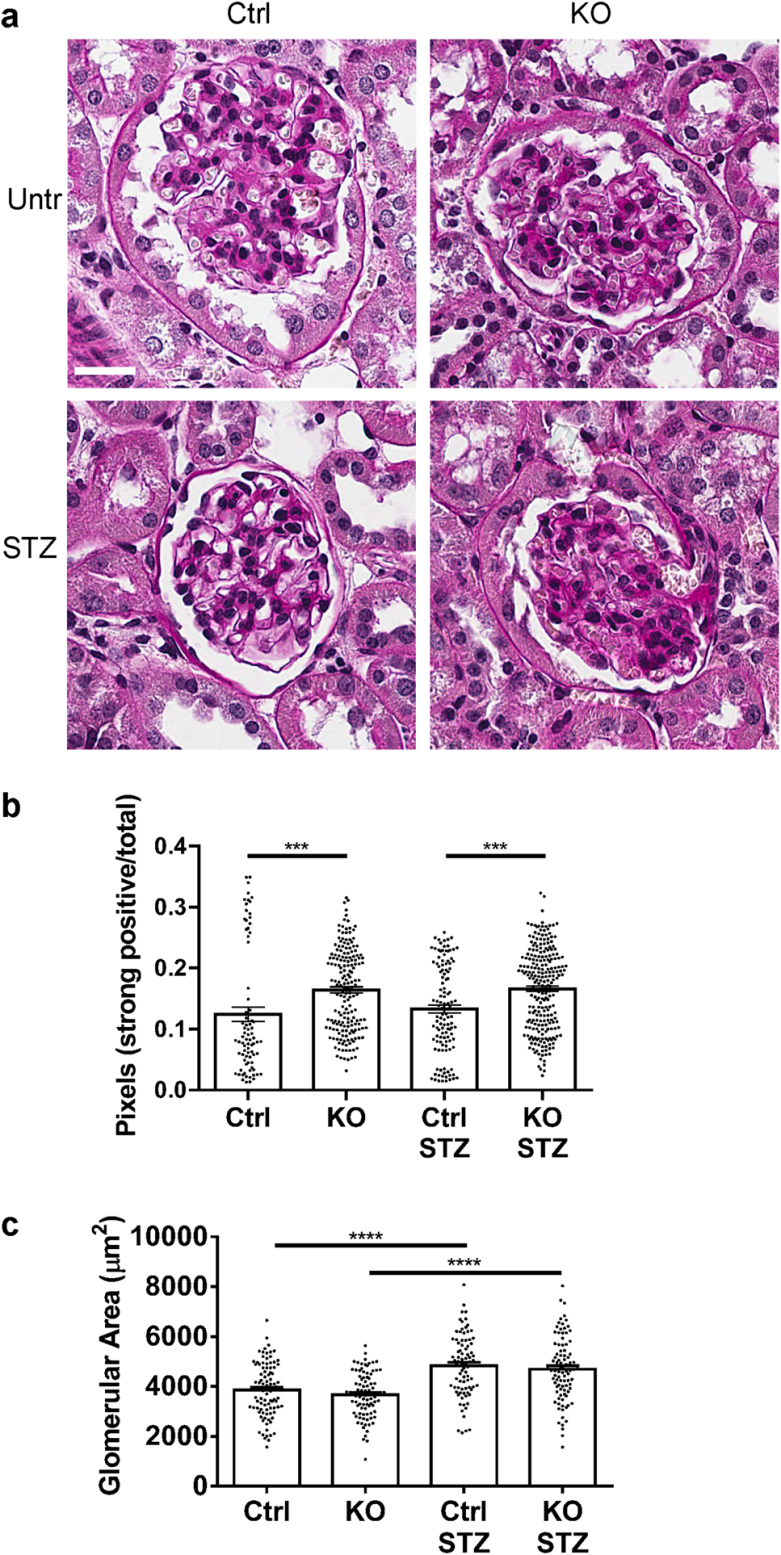
IRE1α KO mice show increased glomerular matrix expansion. Kidney sections were stained with periodic acid-Schiff (**a**), and glomerular matrix expansion was evaluated with a pixel-counting algorithm (**b**). (**a**) Representative photomicrographs; (**b**) Quantification of periodic acid-Schiff staining intensity. (**b**) Both diabetic (STZ-treated) and untreated IRE1α KO mice showed glomerular matrix expansion, compared to control. (**c**) Diabetic (STZ-treated) control and IRE1α KO mice showed greater glomerular cross-sectional areas, compared to untreated groups. Bar = 25 µm. 20 glomeruli in 4–9 mice per group were analyzed (ANOVA). ***P < 0.001, ****P < 0.0001. Other comparisons are not statistically significant.

The baseline urine albumin/creatinine ratio was similar in control and IRE1α KO mice (Fig. 1). These values increased modestly over 6 months. Urine albumin/creatinine increased over 6 months in diabetic control mice; however, albuminuria was significantly exacerbated in diabetic KO mice (Fig. 1). Some variability in urine albumin excretion among the mice within groups was noted. Morphometric quantification of glomerular histology after 6 months of diabetes revealed that untreated and diabetic KO mice displayed increased glomerular extracellular matrix expansion compared with untreated and diabetic control mice, respectively (Fig. 2); however, diabetes did not further increase glomerular matrix significantly. In contrast, diabetic control and IRE1α KO mice showed greater glomerular areas, compared to the untreated groups (Fig. 2), which may reflect cell hypertrophy or larger capillary loops associated with increases in glomerular pressure.

**Figure 3:**
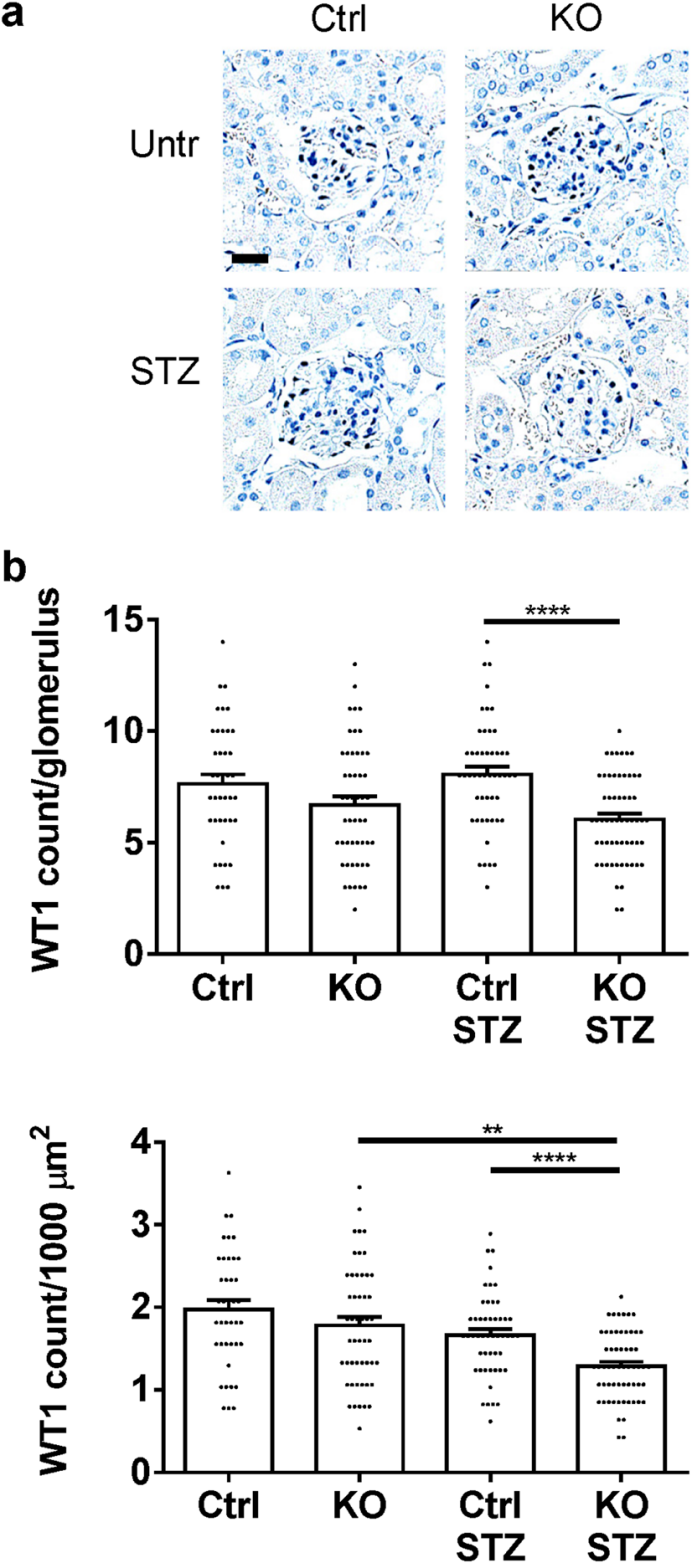
Diabetic (STZ-treated) IRE1α KO mice show a reduction in podocytes (WT1-positive cells). (**a**) Kidney sections were stained with anti-WT1 antibody (representative photomicrographs). (**b**) WT1 counts are presented per glomerulus and per glomerular cross-sectional area. Bar = 25 µm. 10 glomeruli/mouse in 4–6 mice per group were analyzed (ANOVA). **P < 0.01, ****P < 0.0001.

**Figure 4:**
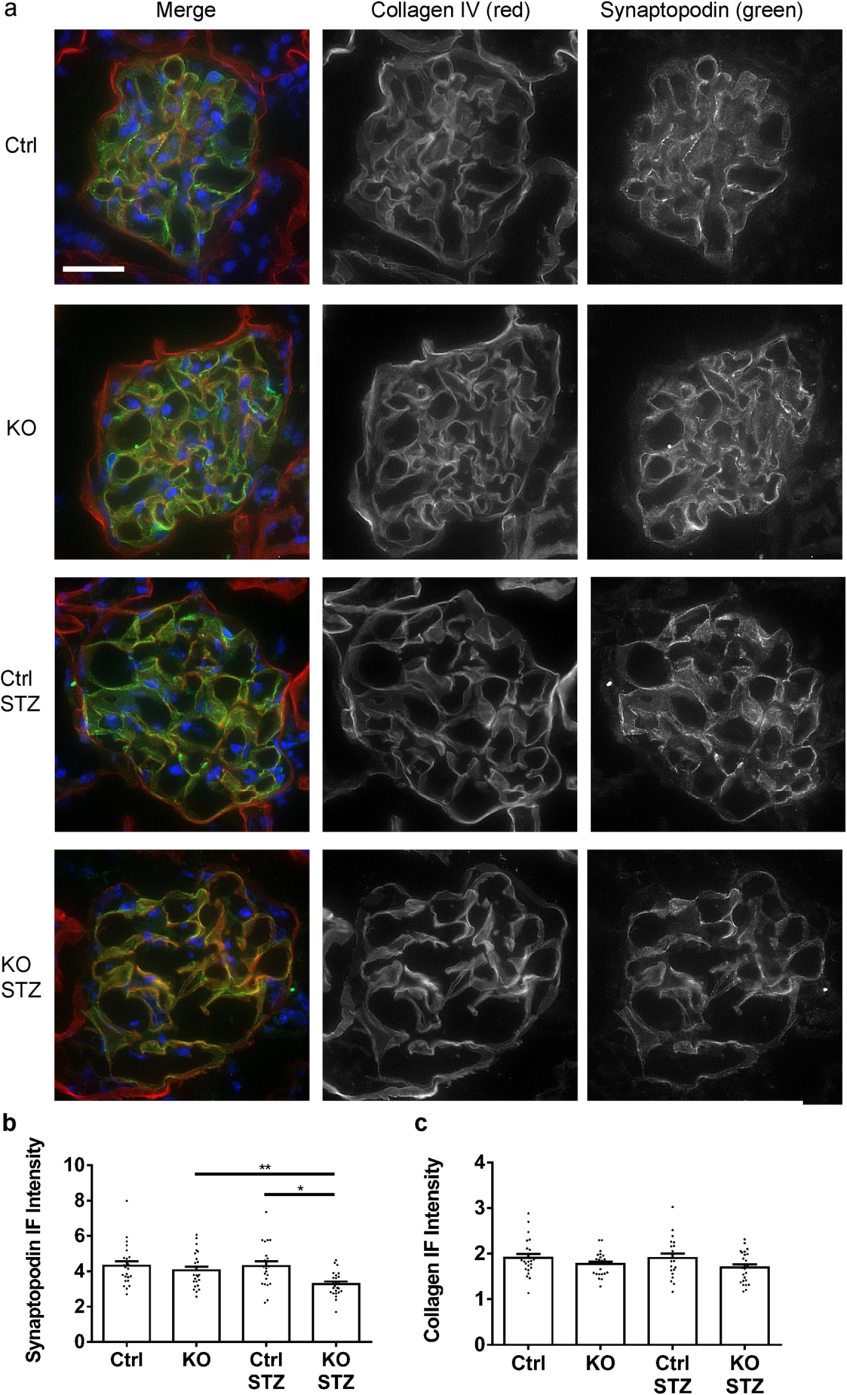
IRE1α KO mice with diabetic nephropathy (STZ) show a reduction in synaptopodin. (**a**) Kidney sections were stained with antibodies to synaptopodin and collagen IV-α5 (representative photomicrographs). Bar = 25 µm. (**b**) and (**c**) Quantification of immunofluorescence intensity. 5–7 glomeruli/mouse in 4 mice per group were analyzed (ANOVA). (**b**) *P < 0.05, **P < 0.01. Control (Ctrl) vs Ctrl STZ is not statistically significant. (**c**) There were no significant changes in collagen staining among groups.

The number of podocytes per glomerulus (WT1-positive cells) was comparable among untreated control, untreated KO and diabetic control mice, but IRE1α KO mice after 6 months of diabetes showed a significant reduction of podocytes (Fig. 3). This was particularly evident when WT1 counts were expressed per glomerular area, which reflects a substantial depletion of podocytes in the larger glomeruli of the diabetic IRE1α KO mice. By analogy, immunofluorescence microscopy showed comparable glomerular expression of the podocyte differentiation marker synaptopodin in untreated control, untreated KO and diabetic control mice, but synaptopodin was reduced significantly in IRE1α KO mice with diabetes (Fig. 4). There were, however, no significant differences in expression of the GBM protein collagen IV-α5. In addition, there were no significant differences in the colocalization of collagen IV-α5 and synaptopodin among the groups (the Pearson correlation coefficients were 0.633 ± 0.062, 0.621 ± 0.087, 0.629 ± 0.087 and 0.644 ± 0.085, respectively).

Immunofluorescence microscopy showed comparable glomerular expression of the podocyte differentiation markers nephrin and podocalyxin among the 4 groups of mice; there was reduced expression of podocalyxin in mice after 6 months of diabetes, which did not reach statistical significance (Supplementary Fig. 2). Immunoblotting confirmed that there were no significant differences in glomerular expression of nephrin, while podocalyxin was reduced significantly in diabetic mice (Supplementary Fig. 3). The differences in statistical significance between the two techniques could be related to distinct methods of tissue preparation, or that antibodies may react differently with their antigens in immunoblot membranes versus tissue sections. Glomerular F-actin (measured by fluorescence of rhodamine-phalloidin) was reduced in untreated IRE1α KO mice compared to untreated control. F-actin was reduced significantly in diabetic control mice compared to untreated control, with further reduction in diabetic IRE1α KO mice that did not reach statistical significance (Supplementary Fig. 4).

### Deletion of IRE1α in podocytes exacerbates ultrastructural glomerular injury in diabetic nephropathy

**Figure 5:**
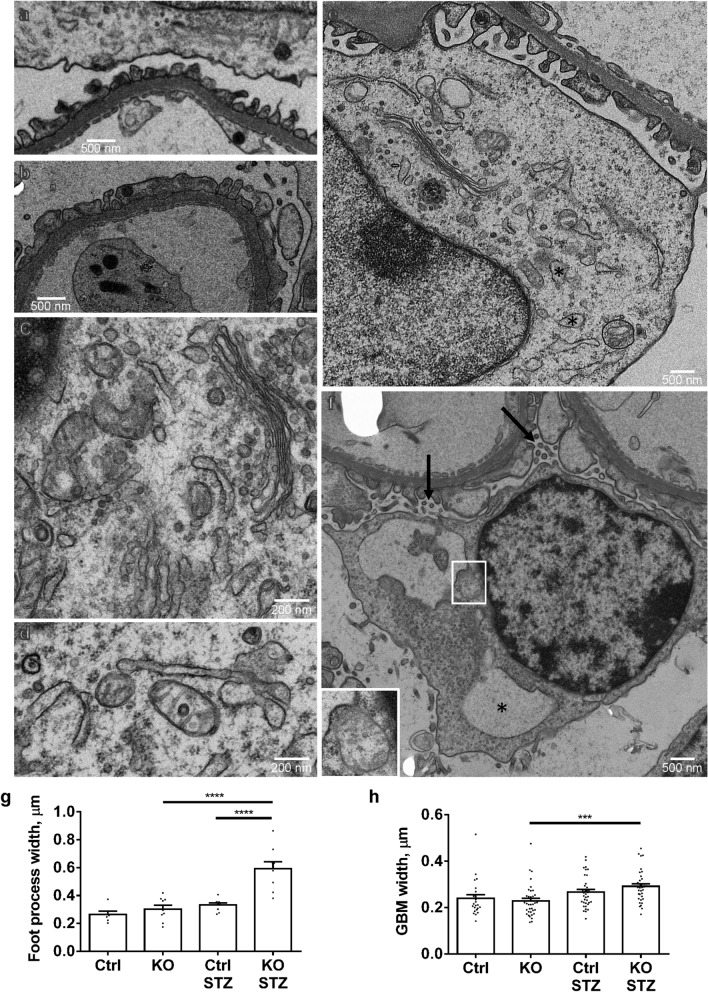
Deletion of IRE1α in podocytes exacerbates podocyte ultrastructural injury in diabetic nephropathy. (**a**) Normal glomerular capillary wall in a control untreated mouse. (**b**) An IRE1α KO diabetic (STZ-treated) mouse shows moderate focal podocyte foot process effacement. (**c**) and (**d**) Organelles, including ER, Golgi and mitochondria appear normal in untreated control (**c**) and untreated IRE1α KO mice (**d**). (**e**) A diabetic control mouse shows normal podocyte foot processes, and most organelles appear normal, but occasional focal swelling of the ER is evident (*).(**f**) An example of microvesiculation of the podocyte plasma membrane (arrows), markedly dilated ER (*) and mitochondrial damage (inset) in a diabetic IRE1α KO mouse. (**g**) and (**h**) Quantification of foot process and GBM width. Diabetic (STZ-treated) IRE1α KO mice show widening of foot processes and GBM. Parameters were measured in 3 glomeruli per mouse, 2 mice in untreated control and 3 mice in the other groups. There are 6–9 measurements of foot process width per mouse. There are 4 measurements of GBM width per capillary loop; 6–9 capillary loops per mouse (ANOVA). ***P < 0.001, ****P < 0.0001.

Kidneys of mice after 6 months of diabetes were examined by electron microscopy. Untreated (non-diabetic) control and IRE1α KO mice showed normal podocyte foot processes and organelles (Fig. 5a,c,d,g). Foot processes demonstrated moderate widening in diabetic IRE1α KO mice, but not in diabetic controls (Fig. 5b,g). Compared with untreated IRE1α KO mice, diabetic IRE1α KO mice demonstrated GBM widening, whereas in control mice, there was modest GBM widening, but the change did not reach statistical significance (Fig. 5h). Organelles, including the ER, Golgi and mitochondria appeared normal in untreated control and untreated IRE1α KO mice (Fig. 5d). In diabetic control mice, most organelles appeared normal, but there was rare focal swelling of the ER (Fig. 5e). Diabetic IRE1α KO mice showed some focal areas of microvillous transformation and vesiculation of the podocyte plasma membranes. Markedly dilated ER, and more prominent mitochondrial damage (disruption of cristae, and loss of matrix density) was also seen, but was not widespread (Fig. 5f). These results imply that IRE1α is not only important in preserving ER structure, but also mitochondrial integrity. Ultrastructural features of podocyte apoptosis were not evident in any glomeruli.

### Deletion of IRE1α in podocytes attenuates the UPR and autophagy in diabetic nephropathy

**Figure 6:**
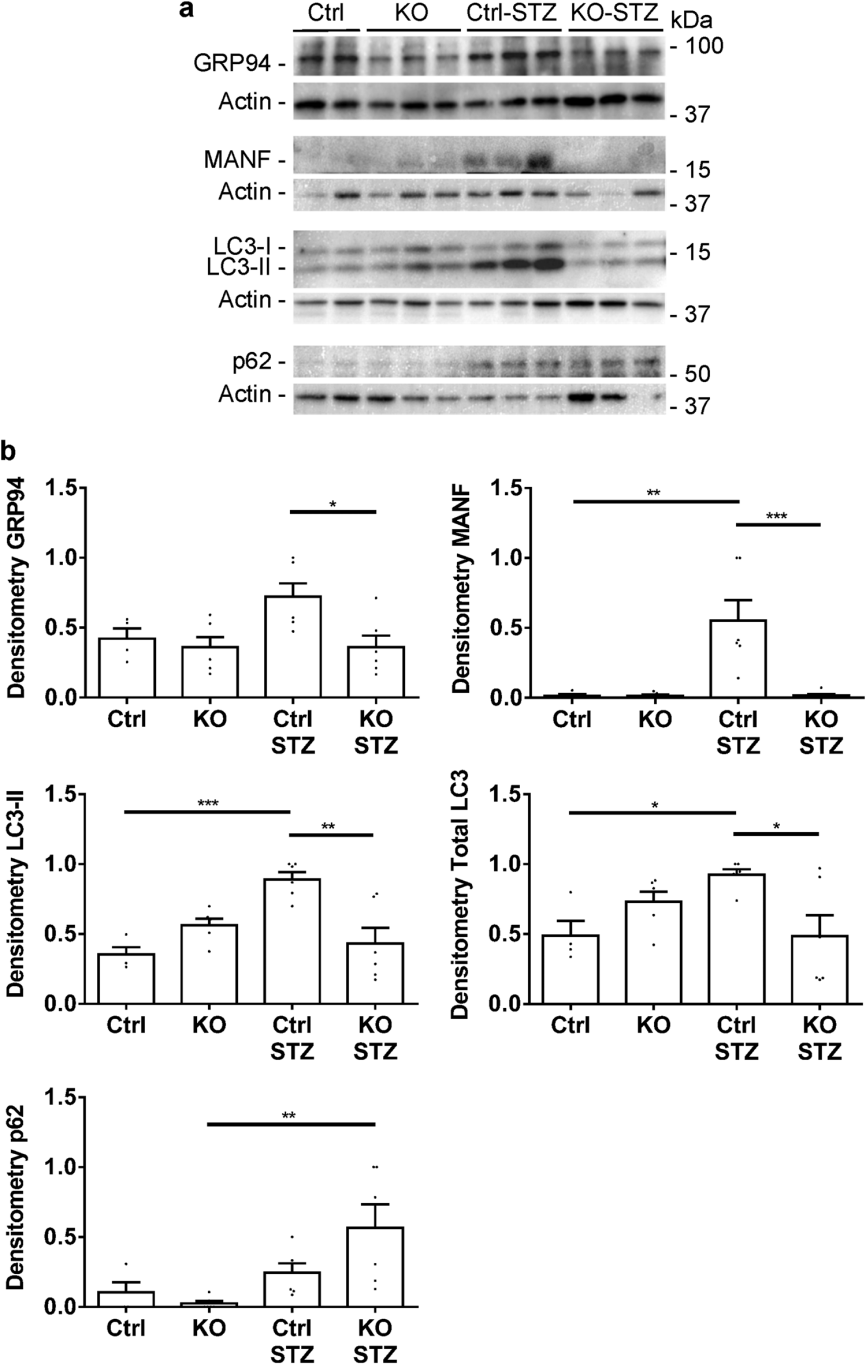
Deletion of IRE1α in podocytes attenuates the UPR and autophagy in diabetic nephropathy. (**a**) Glomerular lysates were immunoblotted with antibodies as indicated (representative immunoblots). (**b**) Signals were quantified by densitometry (values are normalized to the expression of β-actin). The ER chaperones, GRP94 and MANF, as well as LC3-II and total LC3 were increased significantly in diabetic (STZ-treated) control, but not diabetic IRE1α KO mice. p62 was increased in diabetic IRE1α KO mice compared to untreated KO. N = 4 mice in control, 6 in KO, 6 in Ctrl STZ and 6 in KO STZ groups (ANOVA). *P < 0.05, **P < 0.01, ***P < 0.001. The uncropped immunoblots are presented in Supplementary Fig. 9.

In these experiments, we examined the effects of IRE1α and diabetes on the UPR and autophagy. At the end of 6 months of diabetes, glomeruli were isolated from mouse kidneys and lysates were subjected to immunoblotting. Two ER chaperones, including GRP94 and MANF were elevated significantly in diabetic control mice, but not in diabetic IRE1α KO mice (Fig. 6). MANF is an ER chaperone that in GECs/podocytes is exclusively dependent on the IRE1α pathway^15^. Similarly to ER chaperones, LC3-II (a marker of autophagy) and total LC3 levels were increased significantly in diabetic control mice, but not in diabetic IRE1α KO mice. In contrast, p62 (an autophagy substrate) was increased in diabetic IRE1α KO mice, but not in diabetic control mice (Fig. 6), implying reduced degradation of p62 by autophagy in the diabetic IRE1α KO mice. These data indicate that diabetes activated the UPR and autophagy in control mice, but that activation was impaired in IRE1α KO mice. Furthermore, the results suggest that diabetes increased production of LC3 in an IRE1α-dependent manner.

**Figure 7:**
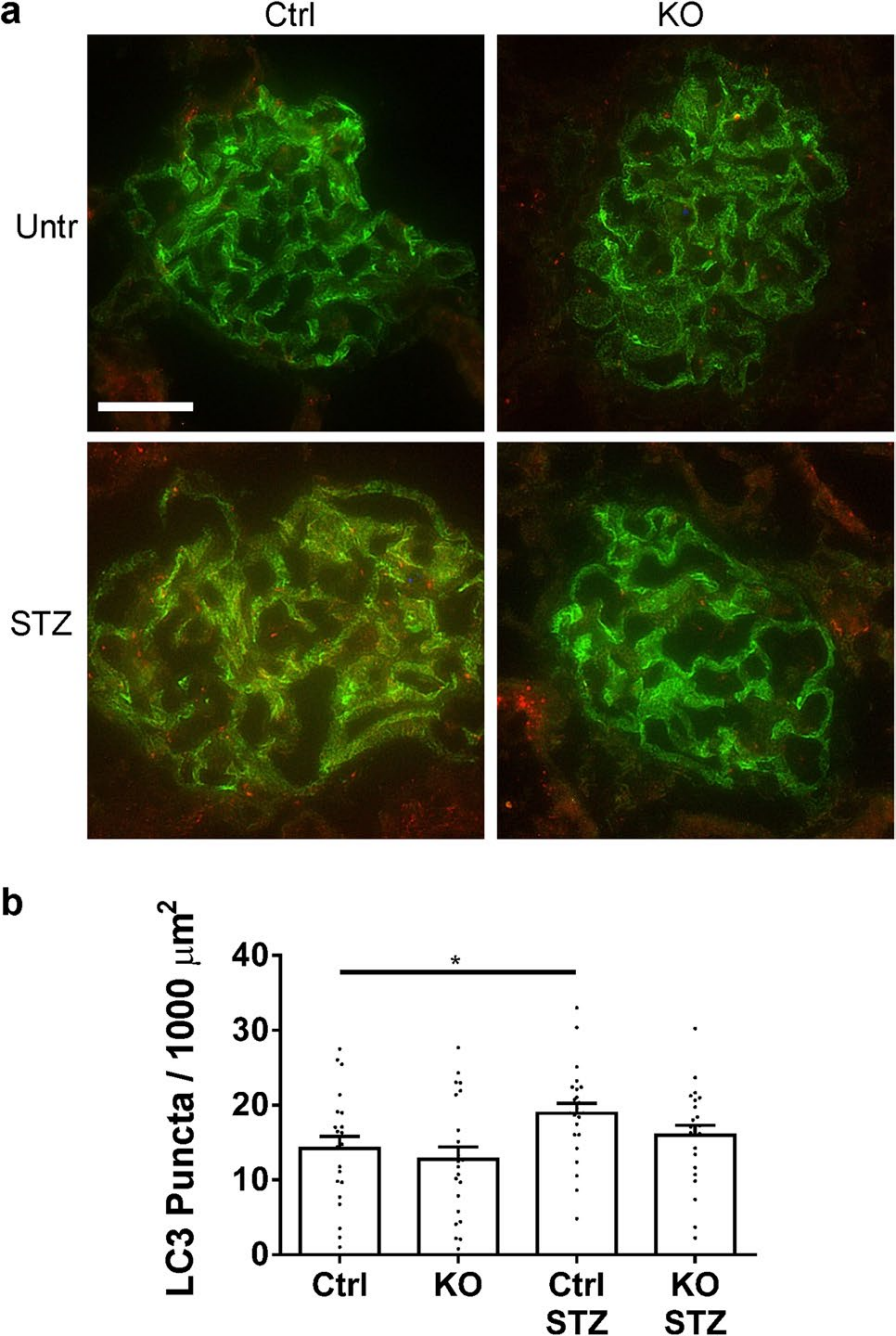
Glomerular LC3 puncta are increased in diabetic (STZ-treated) control mice. (**a**) Kidney sections were stained with antibodies to LC3 (red) and synaptopodin (green; representative photomicrographs). Bar = 25 µm. (**b**) Quantification of puncta. The number of LC3 puncta was increased in diabetic (STZ-treated) control mice, compared to untreated. There was no significant increase in diabetic IRE1α KO mice compared to IRE1α KO control. 5–7 glomeruli/mouse in 4 mice per group were analyzed (ANOVA). *P < 0.05. It should be noted that LC3 puncta generally colocalized with synaptopodin. The grayscale separated channel images are presented in Supplementary Fig. 5.

We also examined formation of glomerular LC3 puncta by immunofluorescence microscopy. LC3 puncta reflect LC3-II in autophagosome membranes^17,28^. Kidney sections were stained with antibodies to LC3 and synaptopodin. The number of LC3 puncta was increased in diabetic control mice, compared to untreated, but there was no significant increase in diabetic IRE1α KO mice (Fig. 7 and Supplementary Fig. 5). These results are in keeping with changes in LC3-II by immunoblotting.

Given that diabetic IRE1α KO mice displayed mitochondrial ultrastructural damage, we examined glomerular expression of PGC1α, a master transcription regulator that stimulates mitochondrial biogenesis^15,35^. By immunoblotting and immunofluorescence microscopy, expression of PGC1α appeared to be lower in diabetic control and diabetic IRE1α KO mice, compared with untreated groups, but differences were not statistically significant in the immunoblotting experiments (Supplementary Figs. 3 and 6).

### Autophagy in GECs

**Figure 8:**
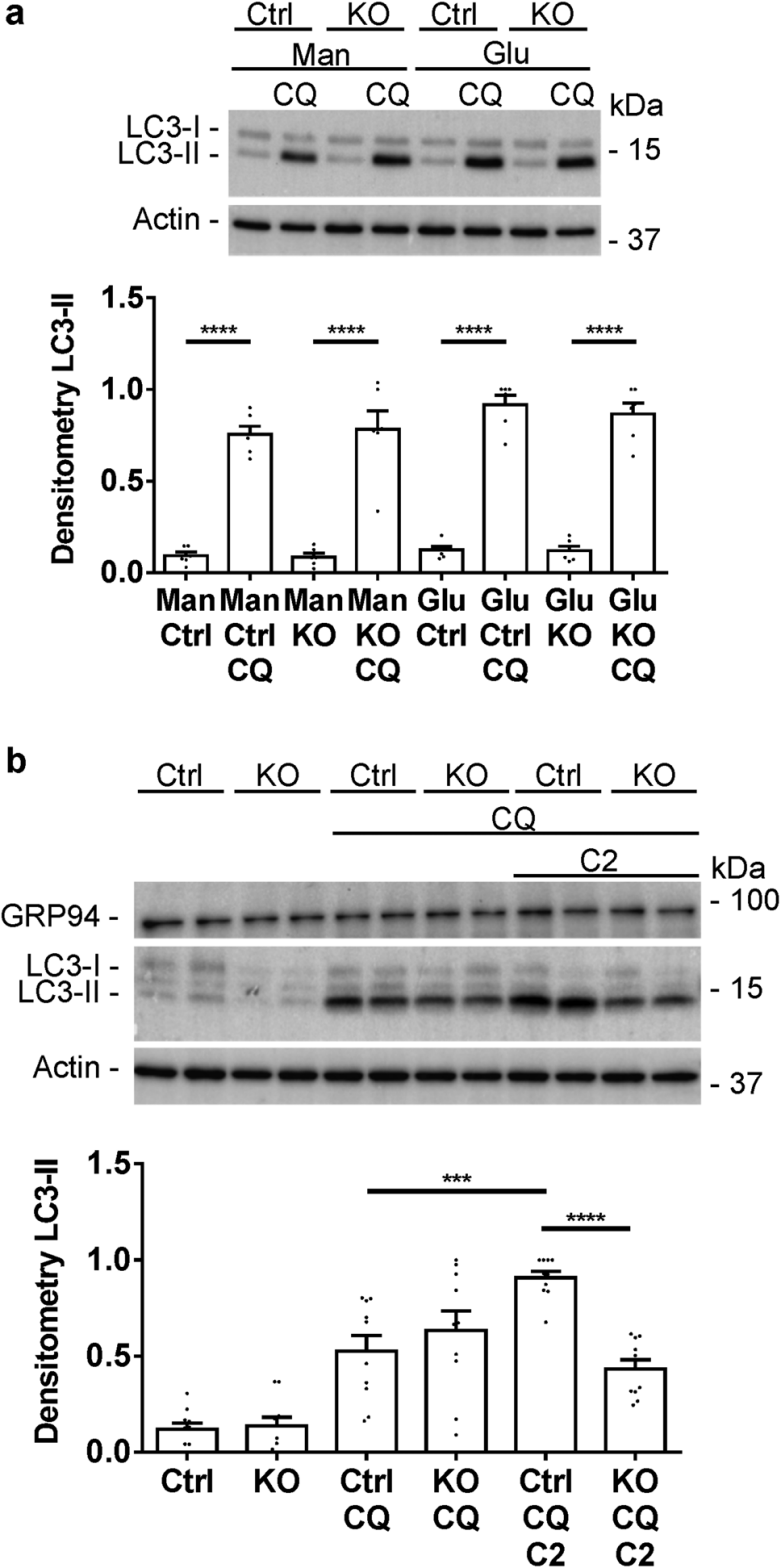
Autophagy in GECs (representative immunoblots and densitometric quantification). (**a**) Control and IRE1α KO GECs were cultured in medium containing 7.8 mM (low) glucose (Glu). Then, medium was switched to 7.8 mM glucose + 28 mM mannitol (Man) or high glucose (36 mM), and cells were treated with or without chloroquine (CQ; 25 µM) for 24 h. LC3-II increased significantly after addition of chloroquine in control and IRE1α KO cells exposed to mannitol or high glucose. There were, however, no significant differences in stimulated LC3-II levels among these 4 groups (ANOVA). 4 experiments performed in duplicate. (**b**) Control and IRE1α KO GECs were untreated, or were incubated with chloroquine (25 µM), or chloroquine + C2-ceramide (C2; 50 µM in 7.8 mM glucose) for 24 h. Chloroquine + C2-ceramide increased LC3-II in control, but not IRE1α KO GECs, compared to chloroquine alone (ANOVA). ***P < 0.001, ****P < 0.0001. There were no differences in GRP94 among the groups. 5 experiments performed in duplicate. The uncropped immunoblots are presented in Supplementary Fig. 9.

In diabetic nephropathy in vivo, autophagy was increased in an IRE1α-dependent manner. Studies to further characterize autophagy were carried out in cultured control and IRE1α KO GECs. In these cell culture experiments, conversion of LC3-I to LC3-II was measured in the presence of chloroquine, which blocks autophagic flux^15,17^. In the first set of experiments, we examined the effect of a high glucose concentration on autophagy. GECs were cultured in medium with 7.8 mM glucose concentration (low glucose). Then, the cells were incubated with either 7.8 mM glucose or were switched to 36 mM glucose (high glucose) for 24 h. Mannitol was employed to control for any potential changes induced by raising osmolality. While addition of chloroquine increased levels of LC3-II, as expected, there was no additional effect of high glucose in both control and IRE1α KO GECs (Fig. 8). In these experiments, incubations with glucose were for 24 h, but similar results (i.e. no effect of high glucose on LC3-II) was also observed at 48 h (results not shown).

In a second set of experiments, GECs were incubated with C2-ceramide (a cell-permeable ceramide analog). Ceramides are a group of bioactive sphingolipids that are elevated in podocytes in diabetes and were reported to mediate albuminuria and podocyte injury in experimental diabetic nephropathy^35–38^. In addition, ceramides were reported to induce ER stress^36^ and autophagy via multiple pathways^39^. In the presence of chloroquine, C2-ceramide stimulated an increase in LC3-II, as well as total LC3, in control GECs, but not in IRE1α KO cells (Fig. 8 and Supplementary Fig. 7). However, in contrast to the stimulation of autophagy, C2-ceramide did not increase the ER chaperones GRP94 or MANF in GECs (Fig. 8 and Supplementary Fig. 7), suggesting that the effect of C2-ceramide on autophagy may not be dependent on activation of the UPR (i.e. the autophagy and ER chaperone pathways may be separate).

### Analysis of human glomerular gene expression in diabetic nephropathy

In an earlier study, we showed that human glomerulopathies demonstrate increases in the expression of glomerular genes that are associated with the UPR and autophagy^16^. In the present study, we examined this in more detail in human diabetic nephropathy, using the JuCKD-Glom (GSE47183) dataset in Nephroseq. This dataset contains 12 patients with diabetic nephropathy (8 male/4 female; mean age 55 years; mean estimated glomerular filtration rate (eGFR) 53 ml/min/1.73 m^2^), who are compared with 21 healthy controls (12 male/9 female; mean age 47 years; mean eGFR 105 ml/min/1.73 m^2^)^16^. Demographic details of these subjects were published previously^16^. First, we analyzed pathways and gene ontology categories based on all genes upregulated in diabetic nephropathy compared with control (Table 1). We found multiple activated pathways associated with the ER, Golgi, ER stress, protein folding and autophagy (Table 1 and Supplementary Table 2). Analysis of downregulated genes yielded only a few pathways (Supplementary Table 2). We also examined if subsets of genes associated with the ER/UPR or autophagy^16^ were increased in diabetic nephropathy. Among a group of ER/UPR and autophagy genes, 65/271 and 77/391, respectively, were increased significantly in diabetic nephropathy, compared with healthy controls (Supplementary Fig. 8 and Supplementary Table 3). Together, these results provide support for the activation of the UPR and autophagy in human diabetic nephropathy.

**Table 1:** Analysis of human glomerular gene expression in diabetic nephropathy in the JuCKD-Glom (GSE47183)^1^ and GSE30122^2^ databases. In diabetic nephropathy, there is activation of pathways and gene ontology categories associated with the ER, UPR and autophagy.

	q-value
Pathway	
Chaperonin-mediated protein folding^1^	0.006992168
Protein folding^1^	0.010681803
Senescence and autophagy in cancer^1^	0.040958965
Gene ontology term name	
Endoplasmic reticulum lumen^1,2^	2.15E − 06
Endoplasmic reticulum part^1,2^	2.68E − 05
Golgi apparatus^1,2^	5.93E − 05
Endoplasmic reticulum^1,2^	0.000271472
Response to endoplasmic reticulum stress^1^	0.007152967
Golgi subcompartment^1^	0.007653156
Lumenal side of endoplasmic reticulum membrane^2^	0.009138649
Golgi apparatus part^1,2^	0.012410582
Golgi membrane^1^	0.0200646
Golgi lumen^2^	0.023906
Endoplasmic reticulum membrane^1^	0.024931078
Endoplasmic reticulum subcompartment^1^	0.028121268
Perinuclear endoplasmic reticulum^2^	0.02955187

Next, we analyzed pathways and gene ontology categories based on changes in glomerular gene expression in a second dataset of patients with diabetic nephropathy (GSE 30122)^34^. This dataset contains 9 patients with diabetic nephropathy (4 male/5 female; mean age 64 years; mean eGFR 31 ml/min/1.73 m^2^), who are compared with 13 healthy controls (8 male/5 female; mean age 51 years; mean eGFR 81 ml/min/1.73 m^2^)^34^. A number of gene ontology categories related to the ER were increased in diabetes (Table 1 and Supplementary Table 4), but changes were not as extensive as in the JuCKD-Glom dataset. There were also some decreased categories, primarily related to the Golgi. It should be noted that this second dataset includes mainly subjects with advanced kidney disease, and the patients showed significant sclerosis on histology and renal impairment. Thus, the results may be less relevant to the pathogenic mechanisms earlier in the course of diabetic nephropathy.

## Discussion

The present study shows that deletion of IRE1α in podocytes exacerbates albuminuria in experimental type I diabetic nephropathy. While albuminuria increased in diabetic control mice, albuminuria was significantly exaggerated in diabetic IRE1α KO mice over a 6-month period. Furthermore, after 6 months of diabetes, the number of podocytes per glomerulus and the expression of synaptopodin was comparable among untreated (non-diabetic) control, untreated IRE1α KO and diabetic control mice, but diabetic IRE1α KO mice showed a significant reduction. Deletion of IRE1α in podocytes exacerbated ultrastructural glomerular injury in diabetic nephropathy. Foot processes and the GBM demonstrated widening in diabetic IRE1α KO mice, but not in diabetic controls. Disruption of the podocyte plasma membranes, significantly dilated ER and prominent mitochondrial damage were evident only in diabetic IRE1α KO mice, although the changes were not widespread. Therefore, in diabetic nephropathy, IRE1α is important in preserving podocyte structure and function, and interestingly, IRE1α preserves not only ER integrity, but also mitochondrial structure. Although diabetic IRE1α KO mice showed widened GBMs, we did not detect changes in glomerular collagen IV expression. Possibly these mice had increases in other collagen isoforms, such as collagen I (interstitial collagen).

Since IRE1α is a transducer of the UPR and was shown to be linked to autophagy^5^, we investigated if these processes were affected in diabetic nephropathy. The ER chaperones GRP94 and MANF, as well as the marker of autophagy LC3-II (and total LC3 levels), were increased in glomeruli of diabetic control mice, but not diabetic IRE1α KO mice. Conversely, the autophagy substrate p62 was increased in diabetic IRE1α KO mice, but not in the diabetic control group. Thus, IRE1α mediated activation of the UPR and autophagy, as well as production of LC3 in diabetes. The apparent increased podocyte injury in IRE1α KO mice is at least in part attributable to impaired UPR and autophagy. Since diabetic IRE1α KO mice showed mitochondrial damage, we examined glomerular expression PGC1α; however, we did not observe consistent changes. Thus, loss of IRE1α did not apparently affect mitochondrial biogenesis, although impaired mitochondrial biogenesis has been observed in diabetic kidneys^35^. The presence of damaged mitochondria, in view of the impaired autophagy, may reflect a disruption of their clearance by mitophagy^12^.

The results of our study, which demonstrates an adaptive/protective role of IRE1α in type I diabetic nephropathy are in keeping with an earlier report^40^. In this earlier study, mice with STZ-induced diabetes were followed for 12 weeks, and deletion of IRE1α in podocytes worsened albuminuria at 2–12 weeks. At 12 weeks, diabetic IRE1α KO mice also showed wider foot processes and GBM, podocyte loss and cleavage of caspase-3 compared with diabetic control mice. The study focused on the expression of alcohol dehydrogenase-1, which was reduced in both untreated and diabetic IRE1α KO mice compared with respective control groups. The authors concluded that the mechanisms of interaction between IRE1α and alcohol dehydrogenase-1 will require further investigation. Parameters of ER stress, the UPR or autophagy were not examined, nor was deletion of IRE1α in podocytes.

A number of other studies have addressed parameters of ER stress in experimental type 1 or 2 diabetes. The results have been variable and these have been reviewed elsewhere^4,22,25^. Hyperglycemia, free fatty acids and advanced glycation end products can induce ER chaperones, sXBP1, C/EBP homologous protein (CHOP; a pro-apoptotic transcription factor) and oxidative stress, and can induce apoptosis in renal cells^22,25^. Outcomes appear to depend on renal cell type and context. In podocytes, diabetic metabolic changes were shown to perturb the balance between the UPR, autophagy and mTOR signaling^22,41,42^.

In STZ diabetes, increased levels of the ER chaperone BiP, phospho-PERK (reflecting PERK activation), CHOP and caspase-12 were reported in glomerular and tubular cells, along with enhanced apoptosis^43^. Moreover, in this model, CHOP KO mice showed less proteinuria, compared with CHOP-replete controls^44^. Recently, combined intervention with CHOP-antisense oligonucleotides and angiotensin-converting enzyme inhibition reduced glomerular and tubular damage in type 2 diabetic nephropathy in db/db mice^45^, in keeping with the result in CHOP KO mice^44^. In db/db mice, ER stress triggered the expression of inflammatory genes^46^. Mice with diabetic nephropathy (either induced by STZ or in db/db mice) showed increased levels of CHOP and activated ATF6 in the renal cortex. In these mice, translocation of sXBP1 into the nucleus was impaired^24^. Lowering of blood glucose or treatment with a chemical chaperone (to improve ER protein folding) normalized sXBP1, ATF6 and CHOP levels, and reduced albuminuria and renal damage associated with diabetes. The severity of diabetic nephropathy and levels of CHOP and ATF6 were exaggerated in podocyte-specific Xbp1-knockout mice and in transgenic mice overexpressing ATF6 in podocytes. The authors concluded that there is distinct regulation of the three ER stress response pathways in diabetic nephropathy, and that loss of XBP1 and induction of ATF6 in podocytes are sufficient to activate a maladaptive UPR that is causally linked to diabetic nephropathy^24^.

Interaction of the UPR with autophagy in diabetes has been addressed in some previous studies^12,47–49^. In one study, the basal level of autophagy in podocytes was reduced in mice with STZ-induced diabetes, as reflected by reductions in LC3-II and other autophagy pathway components^50^. Treatment of STZ-induced diabetic mice with a chemical chaperone attenuated albuminuria, improved glomerular histopathology and restored autophagy^50^. The authors proposed that in diabetic nephropathy, podocytes are subjected to ER stress and autophagy is impaired, whereas restoration of autophagy attenuates podocyte injury. Two other studies made similar conclusions^51,52^. However, deletion of Atg5 autophagy component in podocytes resulted in accelerated podocyte injury and albuminuria in STZ diabetic nephropathy^53^. Interestingly, a similar phenotype developed after deletion of Atg5 in glomerular endothelial cells. Thus, autophagy appeared to be a key protective mechanism in both cell types of the glomerular capillary wall. Recently, it was shown that type 2 diabetes increased expression of the TRPC6 channel in podocytes in mice and reduced autophagy, while inhibition of calpain (a cysteine protease) normalized autophagy and reduced albuminuria^54^. Finally, it should be noted that while a majority of studies have focused on the podocyte, a recent study has demonstrated that CD248 (endosialin) is upregulated in mesangial cells in murine types 1 and 2 diabetic nephropathy^55^. This resulted in impaired glomerular XBP1 splicing by IRE1α and induction of CHOP. Global KO of CD248 attenuated glomerular injury, including albuminuria, supporting the view that communication among glomerular cell types mediates diabetic nephropathy.

Use of chemical chaperones to improve protein folding has provided further evidence for ER stress in diabetic nephropathy. In type 2 diabetic models in db/db mice, chemical chaperones improved blood pressure, fasting plasma glucose levels and insulin tolerance. Chemical chaperones decreased albuminuria, attenuated mesangial expansion and prevented podocyte apoptosis. The effects were paralleled by decreasing levels of ER stress parameters^4,51,52^. These studies demonstrate that chemical chaperones can modulate diabetic nephropathy; however, the extent to which the positive effects of the drugs are related to improvements in glucose control versus renal ER stress has not been established conclusively.

The epidermal growth factor receptor inhibitor erlotinib slowed the progression of STZ-induced diabetic nephropathy in mice, including reduced albuminuria and histological injury. These effects of erlotinib were associated with reduced expression of ER stress markers, and increased expression of autophagy-associated proteins in glomeruli and tubules^56^. There is, however, the possibility to consider that epidermal growth factor receptor inhibitors may be acting by enhancing autophagy, independently of receptor inhibition^16^.

It is important to establish if pathways mediating experimental diabetic nephropathy are relevant to human disease. Using publicly accessible datasets of glomerular gene expression in human kidney biopsies, we found that diabetic nephropathy is associated with activation of multiple pathways involving the ER, Golgi, ER stress, protein folding and autophagy. A substantial number of genes associated with the ER/UPR or autophagy were induced in diabetic nephropathy, compared with healthy controls. A previous study showed that in human diabetic nephropathy kidney biopsies, mRNAs encoding various ER chaperones were elevated^13^. In another study, nuclear localization of sXBP1 was reduced in kidney biopsies of patients with diabetic nephropathy, whereas ATF6 and CHOP were increased, compared with healthy controls^24^. These human studies are limited by relatively small numbers of patients, and there is a lack of uniform criteria for performing kidney biopsies in patients with diabetes; nevertheless, the studies do support activation of the UPR and autophagy.

We used GECs in culture with deletion of IRE1α^15^ to delineate autophagy pathways. Previously, we demonstrated that induction of ER stress in cultured GECs (with tunicamycin) enhances both the UPR and autophagy. Furthermore, tunicamycin increased LC3, Atg5 and Atg7 mRNAs, as well as total LC3 protein, in an IRE1α-dependent manner, consistent with a transcriptional effect of IRE1α-XBP1 on autophagy^15^. In the present study, we examined autophagy in the context of diabetes. Unlike diabetes in vivo, exposure of GECs to hyperglycemia did not stimulate autophagy. We then treated GECs with C2-ceramide, since ceramides are a group of bioactive sphingolipids that are elevated in podocytes in diabetes^35–38^. Ceramides have multiple actions, which may include promoting lipid accumulation, antagonizing mitochondrial function, and inducing ER stress, autophagy and mitophagy^36,37,39,57^. Induction of autophagy by ceramides may be secondary to the UPR^57^, or independent of the UPR, e.g. suppression of mTOR activity, activation of AMP-activated protein kinase via starvation, upregulation of beclin-1, or other mechanisms^39^. In keeping with enhanced autophagy in diabetes in vivo, we found that in cultured GECs, C2-ceramide stimulated an increase in LC3-II and in total LC3 in an IRE1α-dependent manner. However, in contrast to diabetes in vivo, C2-ceramide did not increase GRP94 or MANF in GECs, suggesting that the effect of C2-ceramide on autophagy was independent of the UPR. Earlier, we showed that GECs with deletion or inhibition of IRE1α displayed a defect in autophagosome biogenesis in response to the mTOR inhibitor rapamycin or to glutamine starvation^28^. By analogy to C2-ceramide, rapamycin and glutamine starvation did not or only minimally stimulated sXBP1 or MANF production. These results support the view that IRE1α is able to mediate autophagy not only via sXBP1 and the UPR, but also independently of sXBP1.

Studies on diabetes in cultured GECs have yielded inconsistent results. In one study, high glucose concentrations promoted autophagy in cultured GECs^53^, while in another, levels of autophagy markers were reduced in GECs that were exposed to high glucose^50^. In some studies, cocktails of free fatty acids, angiotensin II and/or cytokines were added to high glucose medium perhaps to better reflect the diabetic milieu^37,38^. A limitation of studies in cultured GECs is that these cells may not accurately reflect diabetic nephropathy in vivo, and more complex cell culture models, e.g. co-culture of cell lines, may be required to address mechanistic questions^58^.

In summary, IRE1α is protective to podocytes in mice with diabetic nephropathy. This is associated with activation of the glomerular UPR and autophagy. The results are consistent with gene expression signatures in human diabetic nephropathy and highlight the potential for therapeutically targeting these pathways.

### Supplementary Information

Supplementary Information.

Supplementary Table S2.

Supplementary Table S3.

Supplementary Table S4.

## Data Availability

Data supporting the findings of this study are available within the article and the supplementary information. The datasets presented in this study can be found in online repositories, which are referenced in the article.
