# Supplementary material for: Deletion of IRE1α in podocytes exacerbates diabetic nephropathy in mice
Source: Sci Rep. 2024 May 22;14:11718. doi: 10.1038/s41598-024-62599-7 (PMC11111796; doi:10.1038/s41598-024-62599-7)

Supplementary Table 1. Composition of K1 medium

50 ml Dulbecco's modified eagle medium (with L-glutamine)
50 ml Nutrient mixture F-10
5 ml NuSerum (Corning)
0.5 ml Hormone mix (below)

Hormone mix

Insulin	5 µg/ml
PGE2	25 ng/ml
T3	0.325 ng/ml
Na_2SeO_3	1.73 ng/ml
Transferrin	5 µg/ml
Hydrocortisone	18.1 ng/ml

Supplementary Figure Legends

Supplementary Figure 1. Blood glucose and body weights of mice. a) STZ induced a rapid increase in blood glucose in both control and IRE1α KO mice. KO STZ vs Ctrl STZ is not significant (ANOVA). b) STZ-treated IRE1α KO mice demonstrated some weight loss compared with untreated KO mice and STZ-treated control. Other differences among groups are not statistically significant. Numbers of animals are presented in the legend to Figure 1.

Supplementary Figure 2. Expression of nephrin and podocalyxin. Kidney sections were stained with antibodies to nephrin (a and b) or podocalyxin (c and d). a and c) Representative photomicrographs; b and d) Quantification of immunofluorescence intensity. Bar = 25 µm. There were no significant differences among groups (ANOVA). 5-7 glomeruli/mouse in 4 mice per group were analyzed.

Supplementary Figure 3. Expression of nephrin, podocalyxin and PGC1α. a) Glomerular lysates were immunoblotted with antibodies as indicated. b-d) Signals were quantified by densitometry (values are normalized to the expression of β-actin). b and d) There were no significant differences in nephrin and PGC1α expression among groups (ANOVA). c) Podocalyxin expression was reduced significantly in Ctrl STZ mice, compared with untreated. Podocalyxin was lower in untreated IRE1α KO mice (not significantly), compared to control, and was not reduced further by STZ. N=4 mice in control, 6 in KO, 6 in Ctrl STZ and 6 in KO STZ groups. **P<0.01.

Supplementary Figure 4. Glomerular F-actin content. a) Kidney sections were stained with FITC-phalloidin, which reflects F-actin (representative photomicrographs). b) Quantification of fluorescence intensity. Bar = 25 µm. F-actin was reduced in untreated IRE1α KO mice compared to control. STZ reduced F-actin in control mice, although there was no additional reduction in IRE1α KO mice. 5-7 glomeruli/mouse in 4 mice per group were analyzed (ANOVA). *P<0.05, ****P<0.0001.

Supplementary Figure 5. Glomerular LC3 puncta. Red and green channels are presented separately as grayscale images. Kidney sections were stained with antibodies to LC3 (red) and synaptopodin (green). See legend to Figure 7. Bar = 25 µm.

Supplementary Figure 6. Expression of PGC1α. a) Kidney sections were stained with anti-PGC1α antibody (representative photomicrographs). b) Quantification of fluorescence intensity. STZ reduced PGC1α expression in control mice, compared to untreated. PGC1α was lower in untreated IRE1α KO mice (not significantly), compared to control, and was not reduced further by STZ. *P<0.05. 5-7 glomeruli/mouse in 4 mice per group were analyzed (ANOVA). Bar = 25 µm.

Supplementary Figure 7. Effects of C2-ceramide on total LC3, GRP94 and MANF. Control and IRE1α KO GECs were incubated with chloroquine (CQ; 25 µM), or chloroquine + C2-ceramide (C2; 50 µM) for 24 h. See representative immunoblot in Figure 8b. a) Chloroquine + C2-ceramide increased total LC3 in control, but not IRE1α KO GECs, compared to chloroquine alone (ANOVA). ***P<0.001, ****P<0.0001. b-d) C2-ceramide did not change expression of GRP94 (b) or MANF (c and d). a, b and d show densitometric quantification; c is a representative immunoblot.

Supplementary Figure 8. Glomerular gene expression in human diabetic nephropathy. The figure presents ER/UPR and autophagy genes that are increased in diabetic nephropathy compared with healthy controls (fold-increase above control). All genes are increased significantly ($P<0.05$, hypergeometric test, corrected for multiple comparisons).

Supplementary Figure 9. Uncropped immunoblots.

Supplementary Figure 1

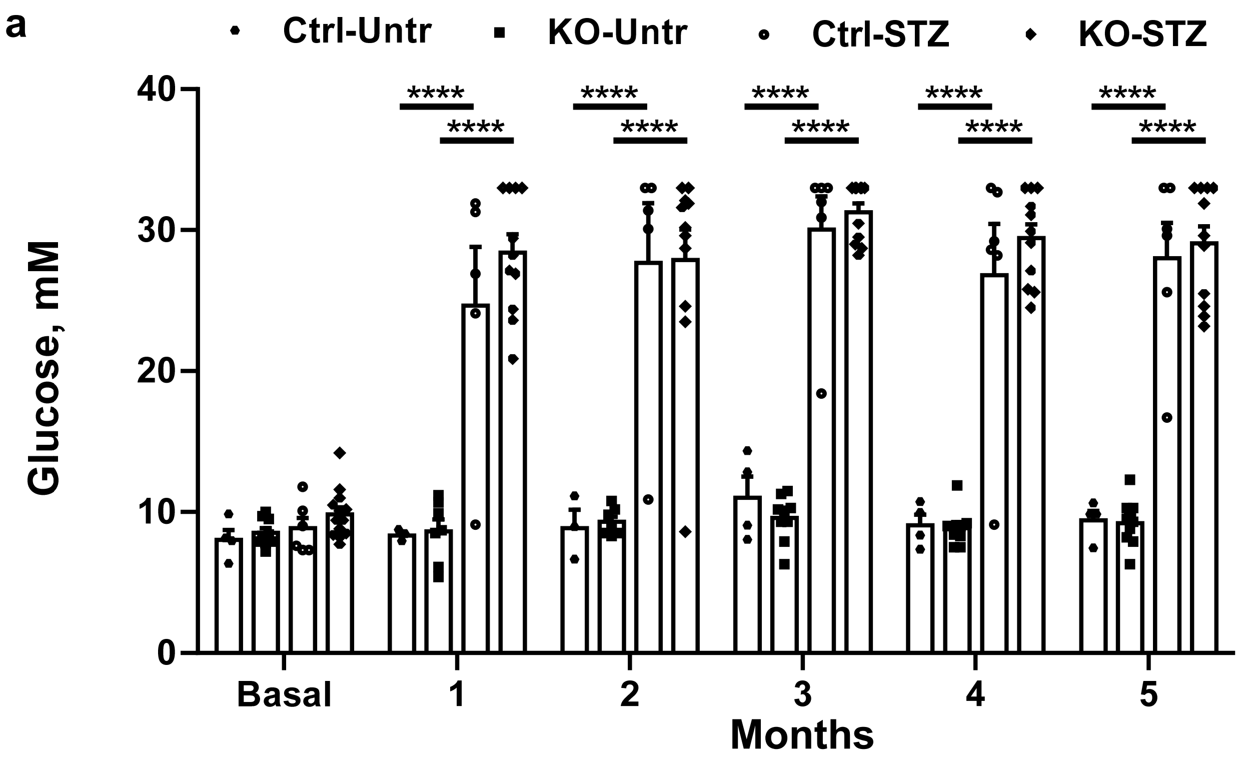

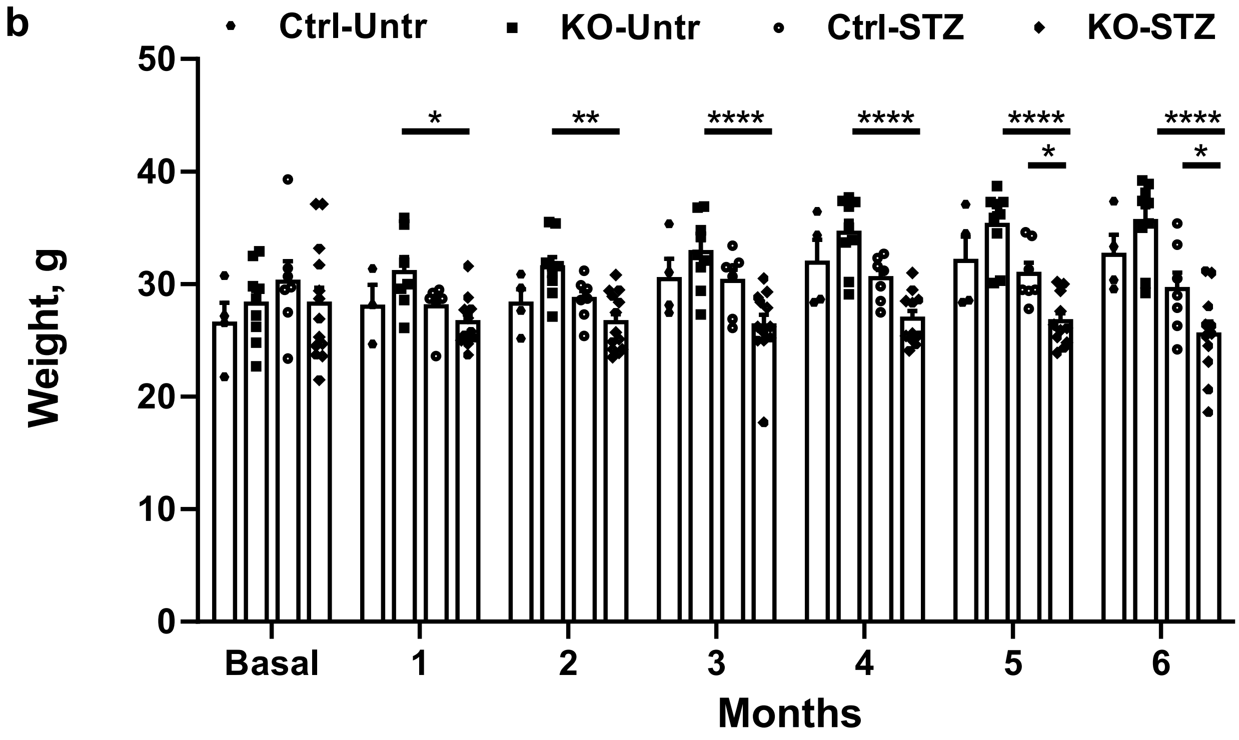

Supplementary Figure 2

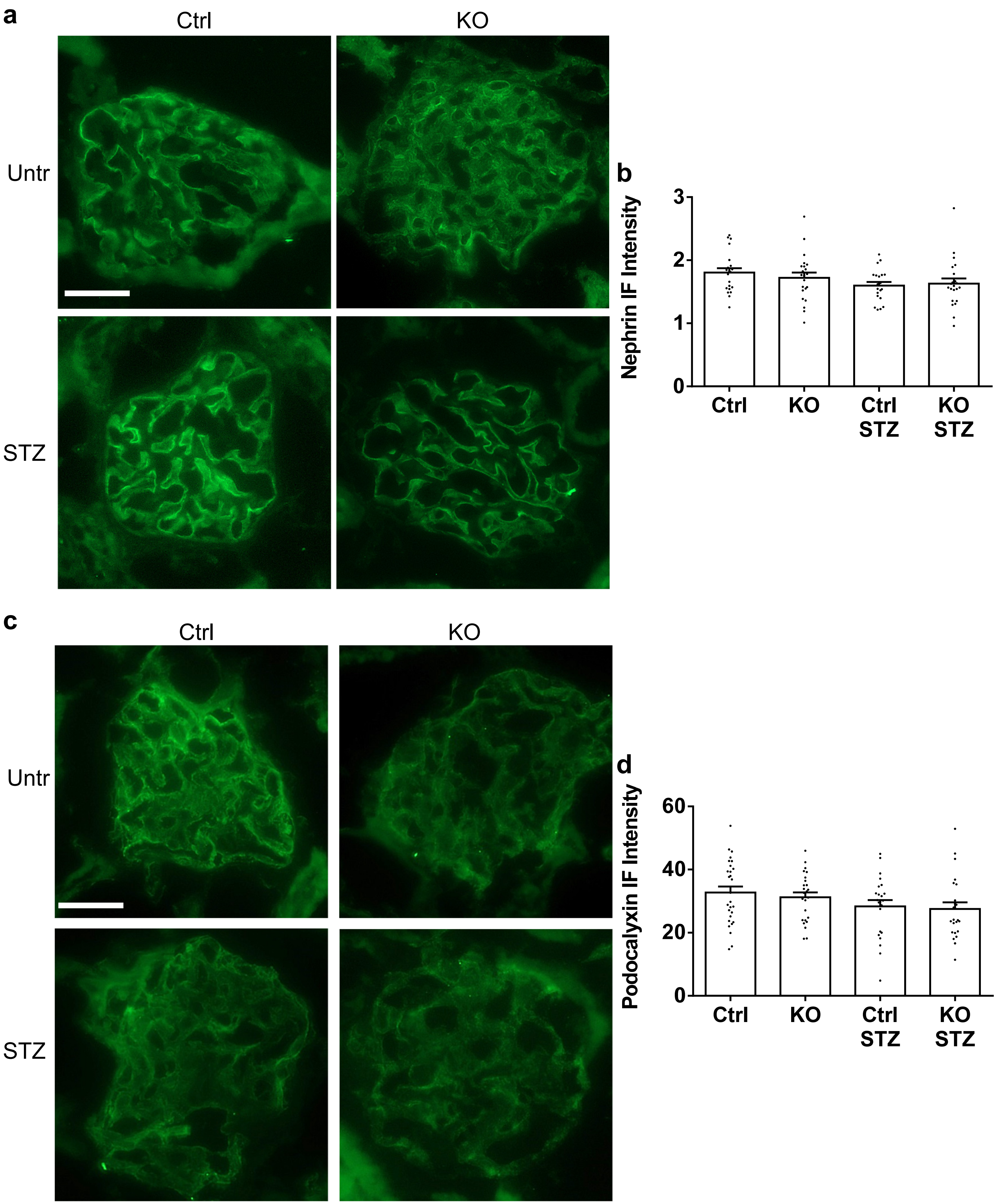

Supplementary Figure 3

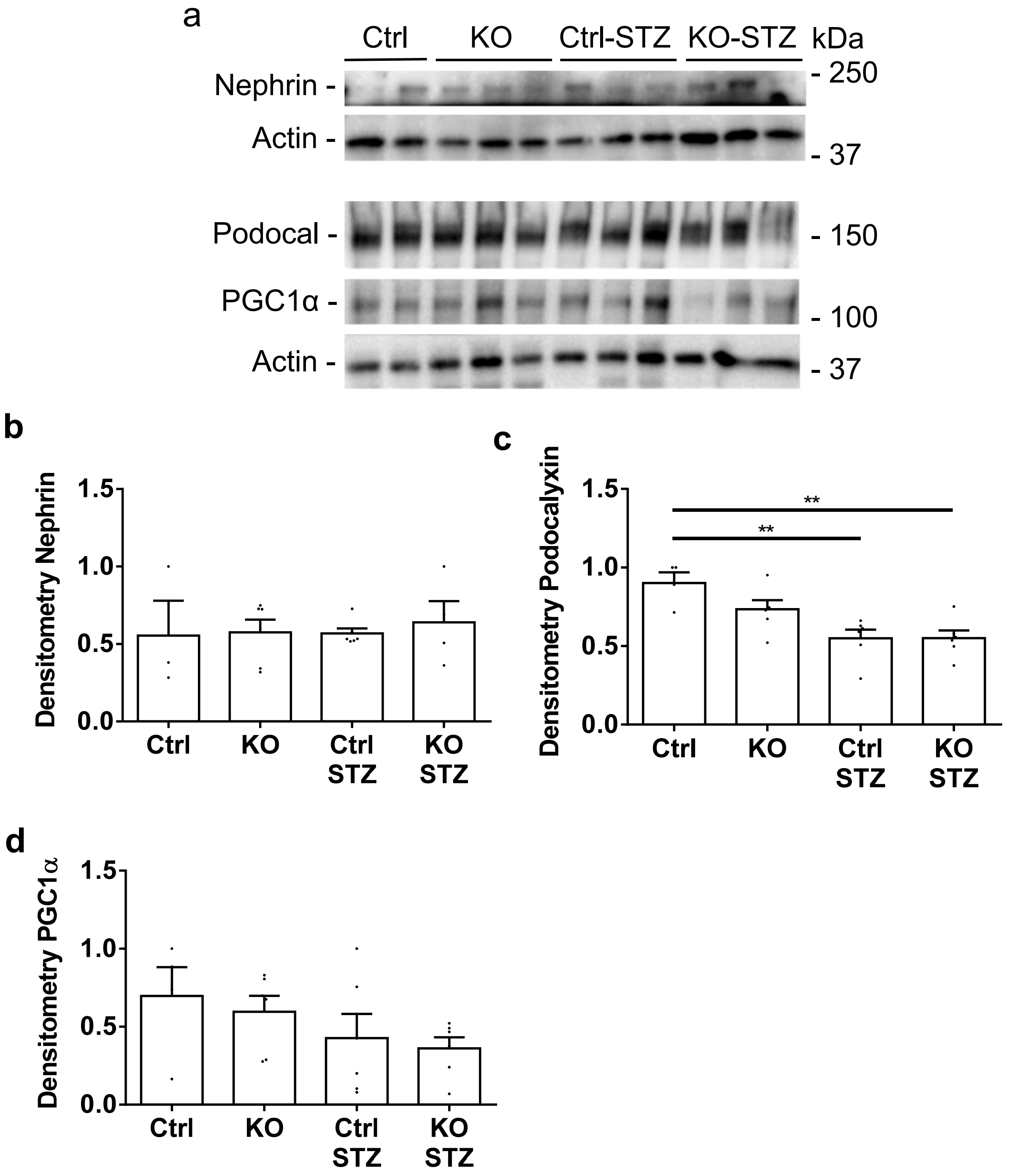

Supplementary Figure 4

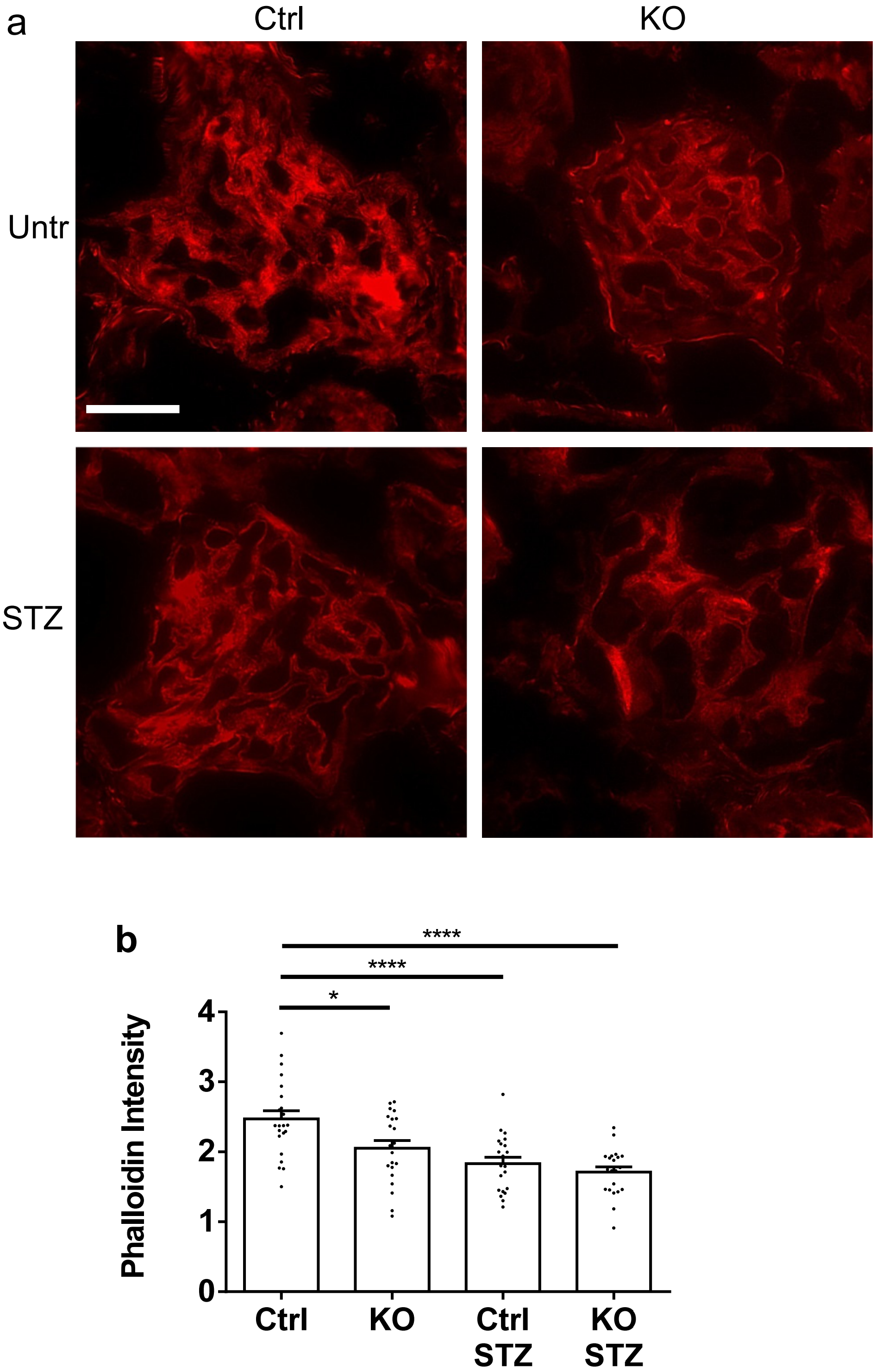

Supplementary Figure 5

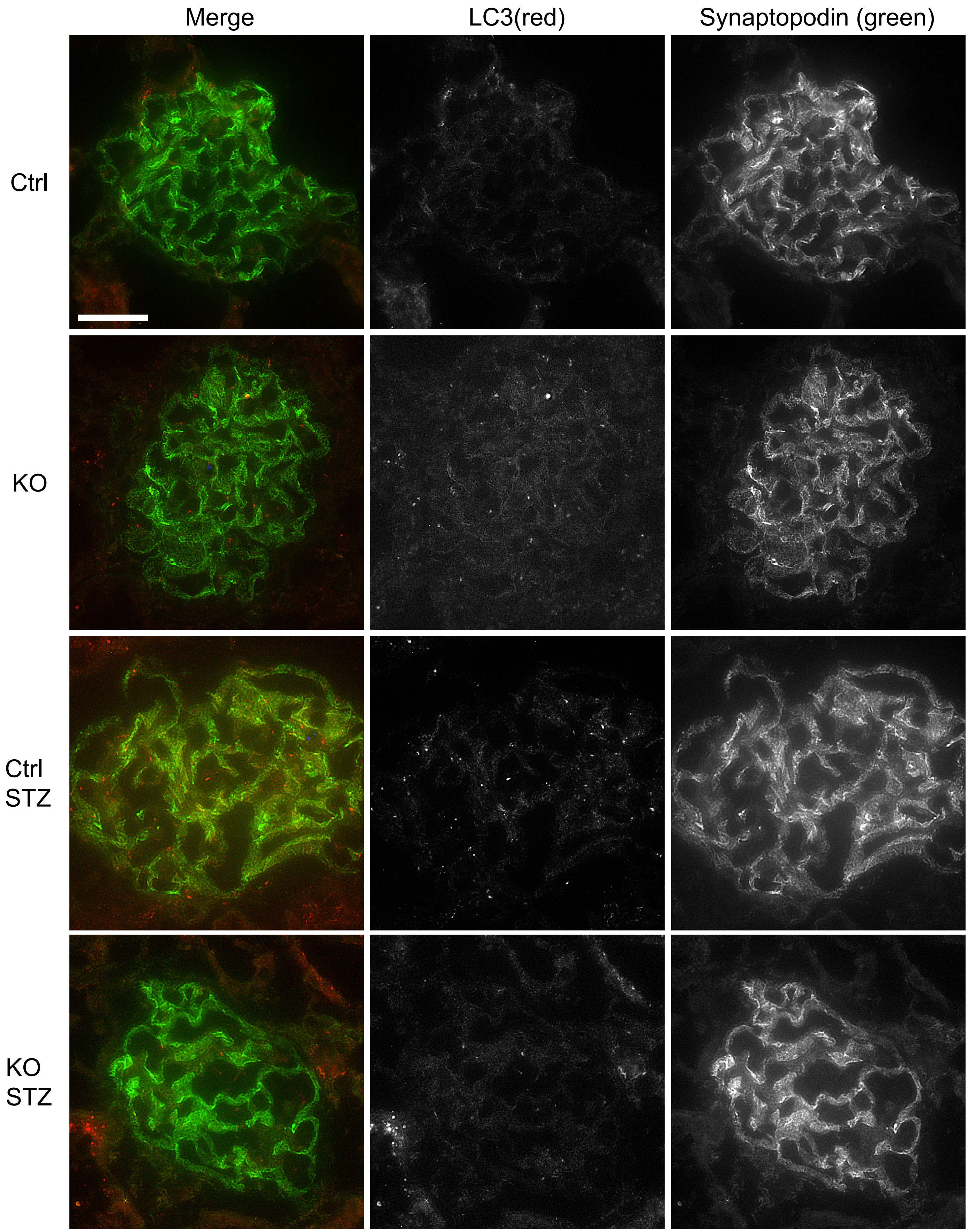

Supplementary Figure 6

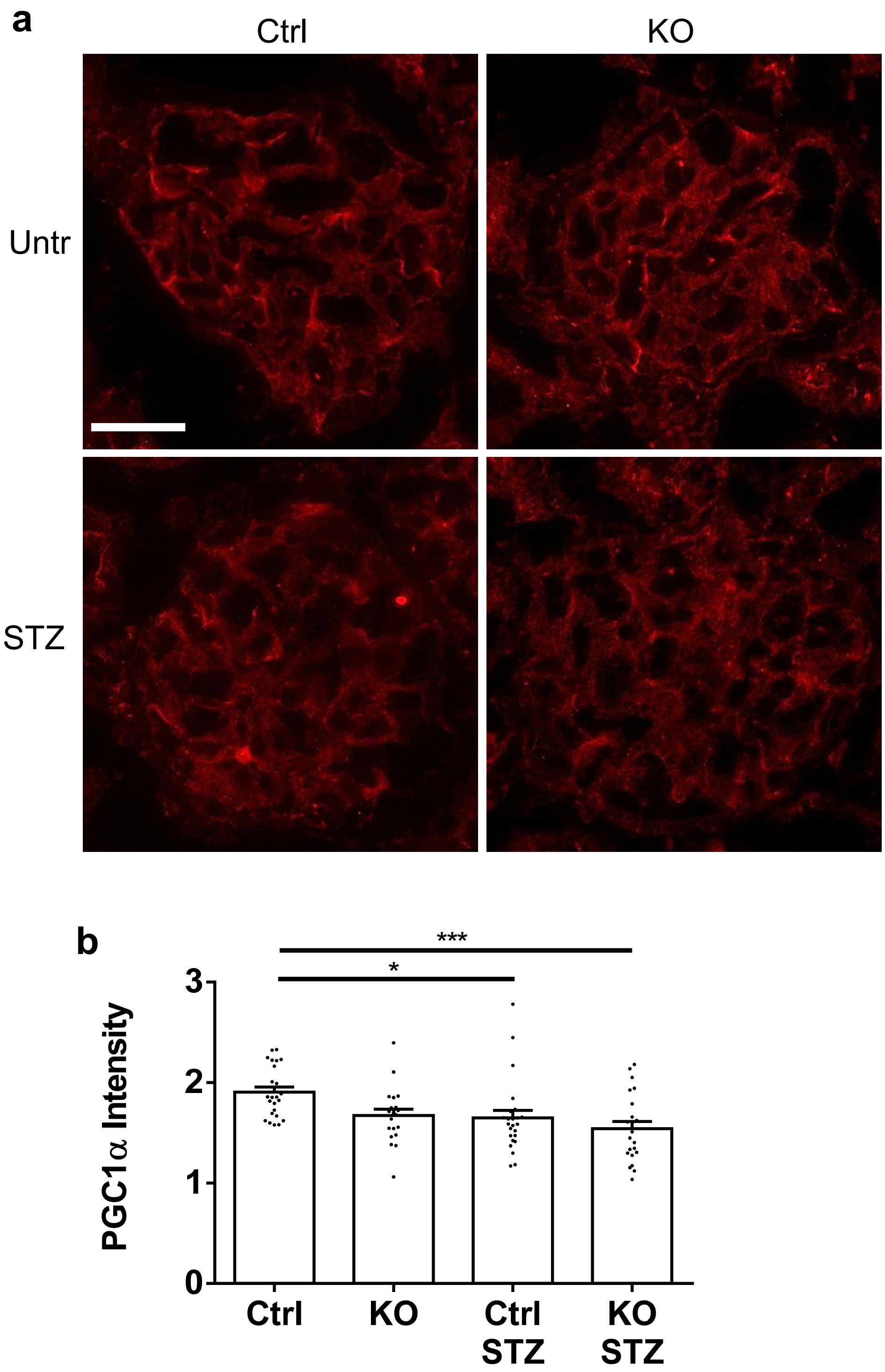

Supplementary Figure 7

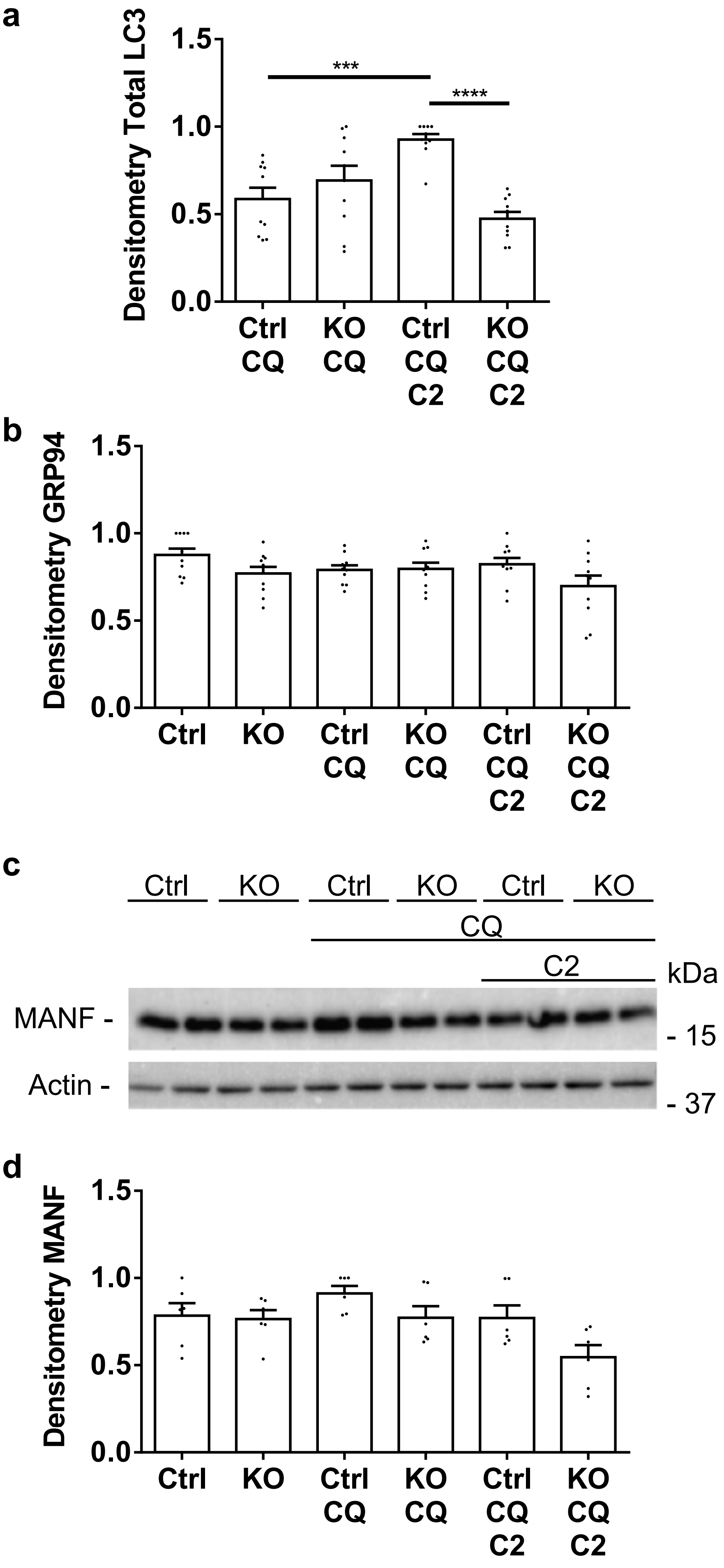

Supplementary Figure 8

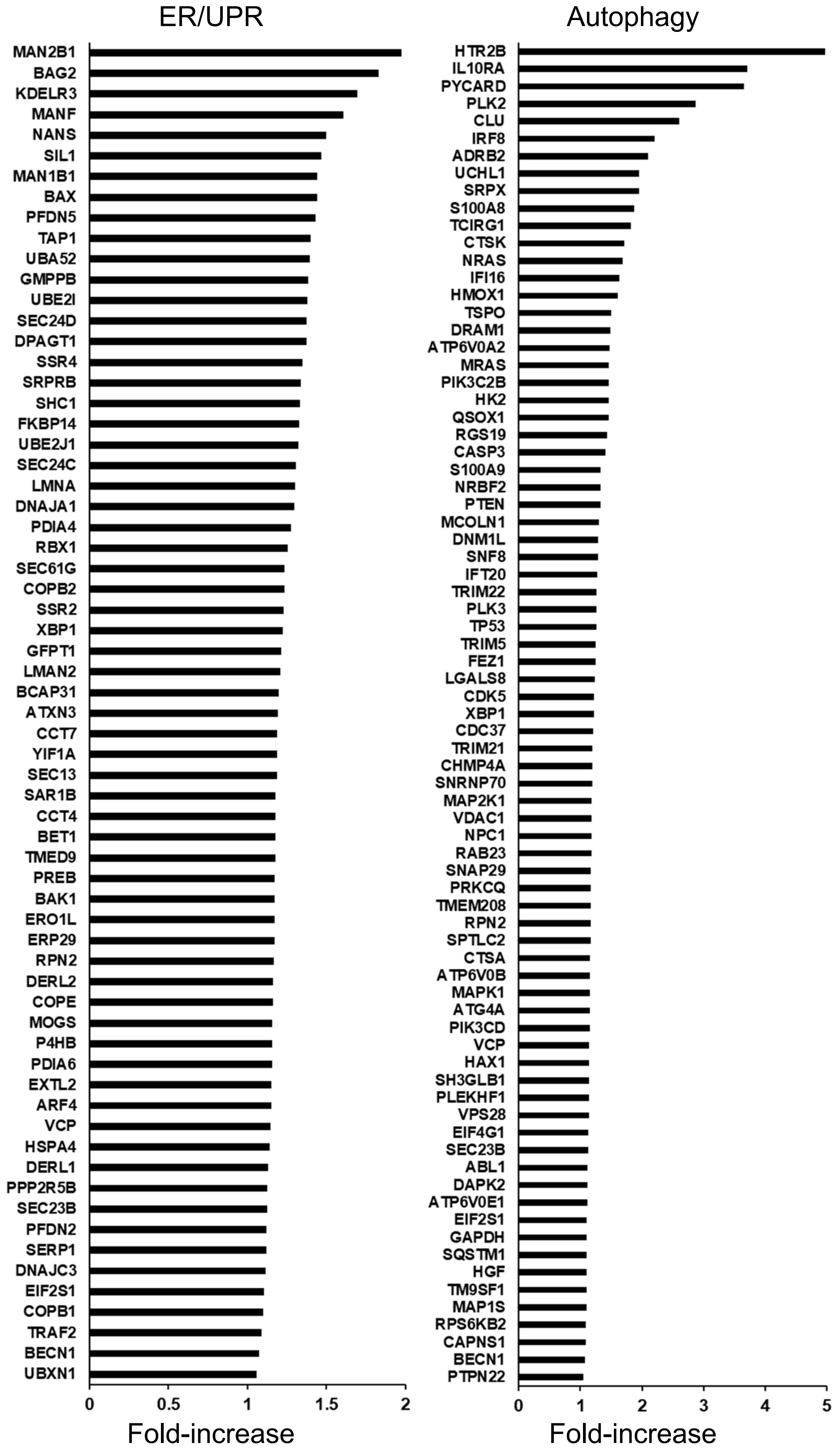

Supplementary Figure 9 – Uncropped Blots

Figure 6

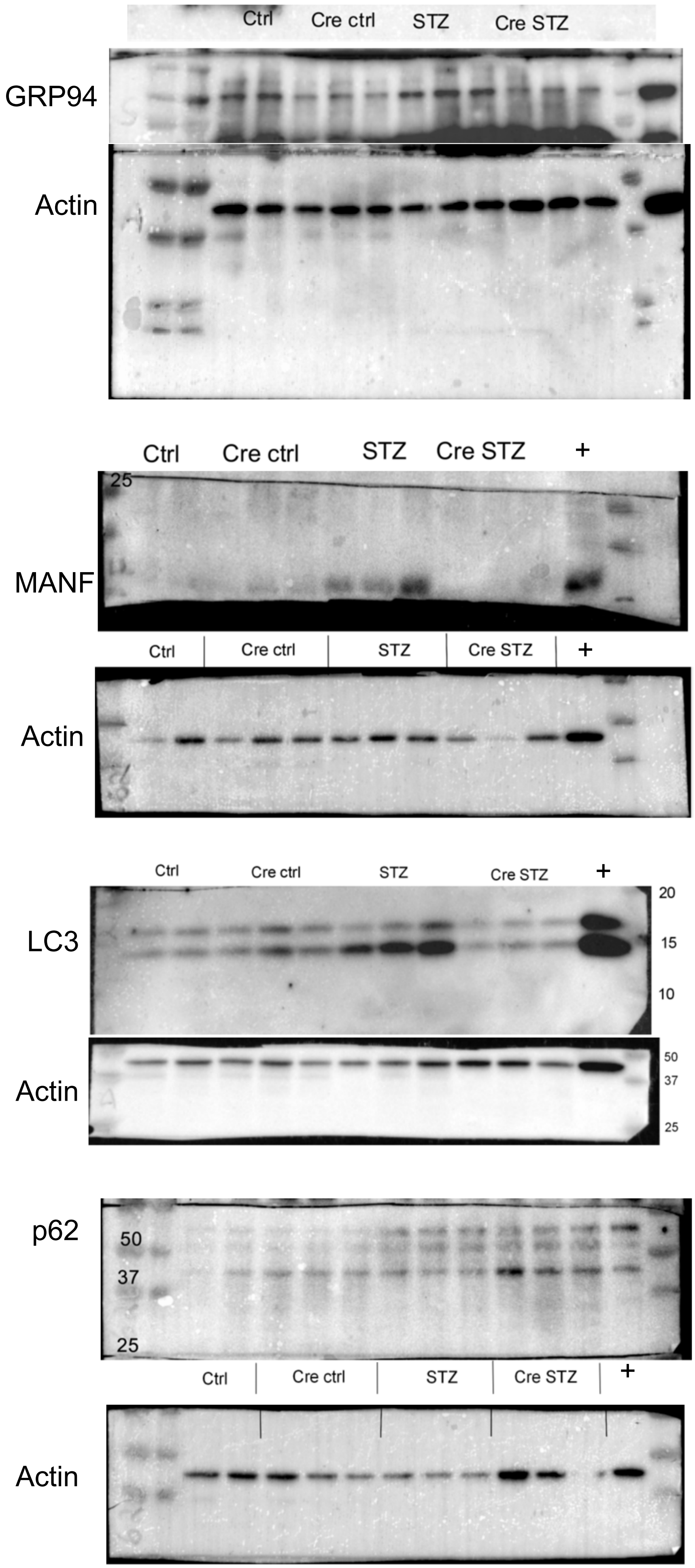

Supplementary Figure 9 – Uncropped Blots, continued

Figure 8

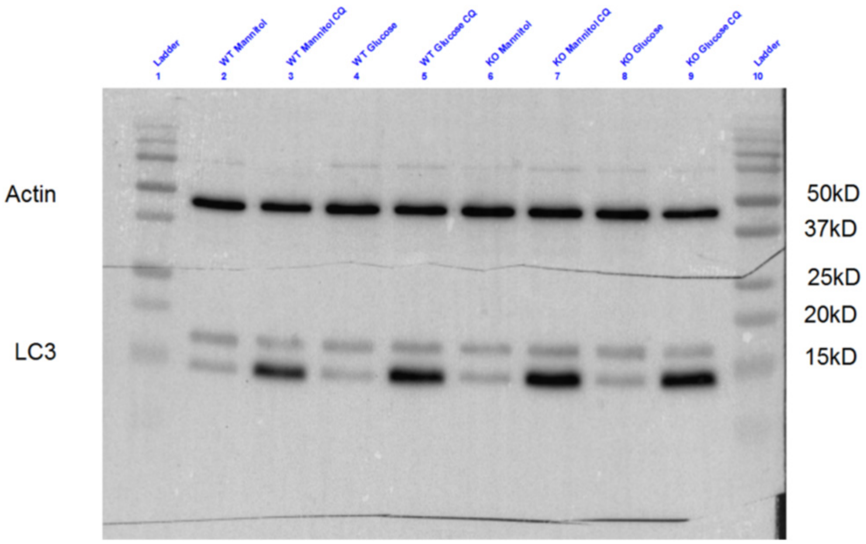

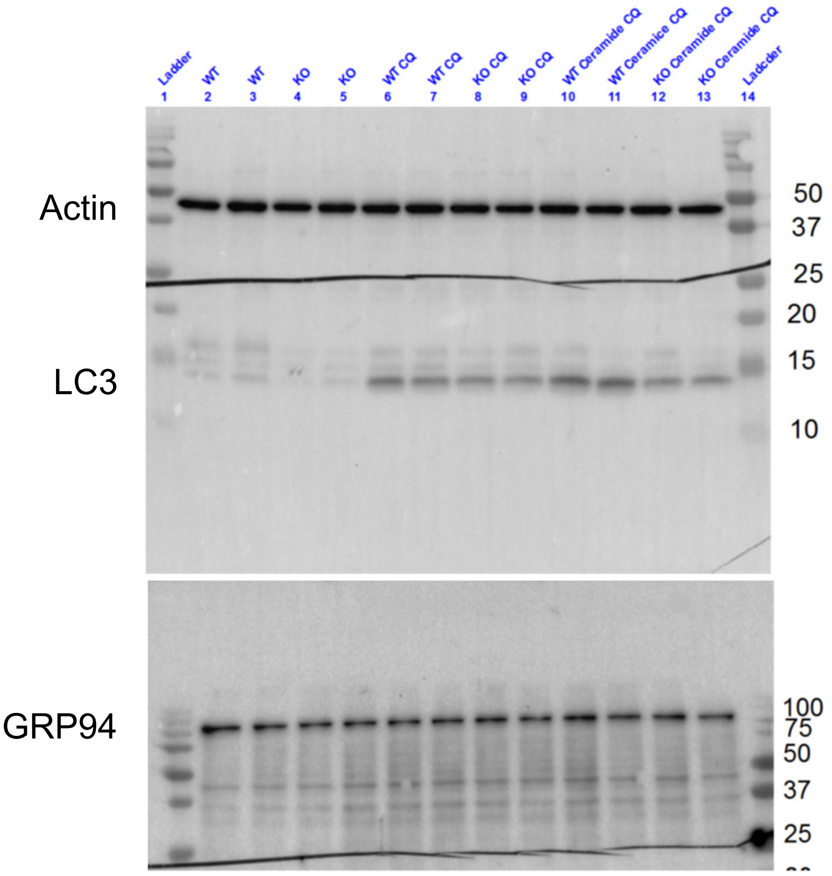

Supplement: Supplementary file 1 — Supplementary Information. [file 41598_2024_62599_MOESM1_ESM.pdf]
